# Plasma proteomic signature of human longevity

**DOI:** 10.1111/acel.14136

**Published:** 2024-03-05

**Authors:** Xiaojuan Liu, Gisli Thor Axelsson, Anne B. Newman, Bruce M. Psaty, Robert M. Boudreau, Chenkai Wu, Alice M. Arnold, Thor Aspelund, Thomas R. Austin, Julius M. Gardin, Kristin Siggeirsdottir, Russell P. Tracy, Robert E. Gerszten, Lenore J. Launer, Lori L. Jennings, Vilmundur Gudnason, Jason L. Sanders, Michelle C. Odden

**Affiliations:** ^1^ Department of Epidemiology and Population Health Stanford University School of Medicine Stanford California USA; ^2^ Faculty of Medicine University of Iceland Reykjavik Iceland; ^3^ Icelandic Heart Association Kopavogur Iceland; ^4^ Department of Epidemiology University of Pittsburgh Pittsburgh Pennsylvania USA; ^5^ Cardiovascular Health Research Unit, Department of Medicine University of Washington Seattle Washington USA; ^6^ Cardiovascular Health Research Unit, Department of Epidemiology University of Washington Seattle Washington USA; ^7^ Cardiovascular Health Research Unit, Department of Health Systems and Population Health University of Washington Seattle Washington USA; ^8^ Global Health Research Center Duke Kunshan University Kunshan China; ^9^ Department of Biostatistics University of Washington Seattle Washington USA; ^10^ Department of Epidemiology University of Washington Seattle Washington USA; ^11^ Division of Cardiology, Department of Medicine Rutgers New Jersey Medical School Newark New Jersey USA; ^12^ Janus Rehabilitation Reykjavik Iceland; ^13^ Department of Pathology and Laboratory Medicine, The Robert Larner M.D. College of Medicine University of Vermont Burlington Vermont USA; ^14^ Department of Biochemistry, The Robert Larner M.D. College of Medicine University of Vermont Burlington Vermont USA; ^15^ Division of Cardiovascular Medicine, Beth Israel Deaconess Medical Center Harvard Medical School Boston Massachusetts USA; ^16^ Laboratory of Epidemiology and Population Sciences, Intramural Research Program National Institute on Aging Bethesda Maryland USA; ^17^ Novartis BioMedical Research Cambridge Massachusetts USA; ^18^ Vertex Pharmaceuticals Inc Boston Massachusetts USA; ^19^ Geriatric Research Education and Clinical Center VA Palo Alto Health Care System Palo Alto California USA

**Keywords:** aging, longevity, proteomics

## Abstract

The identification of protein targets that exhibit anti‐aging clinical potential could inform interventions to lengthen the human health span. Most previous proteomics research has been focused on chronological age instead of longevity. We leveraged two large population‐based prospective cohorts with long follow‐ups to evaluate the proteomic signature of longevity defined by survival to 90 years of age. Plasma proteomics was measured using a SOMAscan assay in 3067 participants from the Cardiovascular Health Study (discovery cohort) and 4690 participants from the Age Gene/Environment Susceptibility‐Reykjavik Study (replication cohort). Logistic regression identified 211 significant proteins in the CHS cohort using a Bonferroni‐adjusted threshold, of which 168 were available in the replication cohort and 105 were replicated (corrected *p* value <0.05). The most significant proteins were GDF‐15 and N‐terminal pro‐BNP in both cohorts. A parsimonious protein‐based prediction model was built using 33 proteins selected by LASSO with 10‐fold cross‐validation and validated using 27 available proteins in the validation cohort. This protein model outperformed a basic model using traditional factors (demographics, height, weight, and smoking) by improving the AUC from 0.658 to 0.748 in the discovery cohort and from 0.755 to 0.802 in the validation cohort. We also found that the associations of 169 out of 211 proteins were partially mediated by physical and/or cognitive function. These findings could contribute to the identification of biomarkers and pathways of aging and potential therapeutic targets to delay aging and age‐related diseases.

## Graphical abstract

A common set of plasma proteins is associated with longevity and could be used to better predict survival to age 90. The protein‐longevity associations vary depending on the survival phenotype used to define them and these associations are differently mediated by multiple functional measurements. These findings could contribute to the identification of biomarkers and pathways of aging which can be prioritized for translation in geroscience trials.

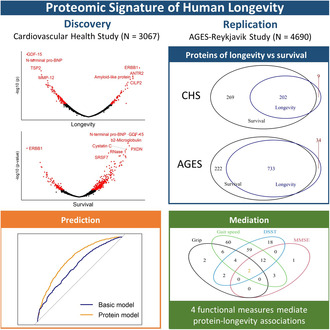

**Abbreviations**
3MSE: modified mini‐mental state examination
ACME: average causal mediation effect
AGES: Reykjavik, Age Gene/Environment Susceptibility‐Reykjavik
ANML: adaptive normalization by maximum likelihood
AUC: area under the receiver operating characteristic curve
CHS: Cardiovascular Health Study
CI: confidence interval
DSST: digit symbol substitution test
HR: hazard ratio
IQR: Interquartile range
LASSO: least absolute shrinkage and selection operator
MSE: mean squared error
OR: odds ratio
ROC: receiver operating characteristic
SD: standard deviations
SOMA: slow off‐rate modified aptamers

## 1 INTRODUCTION

The geroscience hypothesis posits that aging is malleable and delaying the process of aging may concomitantly delay the onset and/or severity of many age‐related diseases and thus increase healthspan (Kennedy et al., 2014; Sierra, 2016). This hypothesis highlights the importance of understanding the basic biology of aging and, in particular, identifying central drivers of the aging process which are strongly associated with disease progression and longevity (López‐Otín et al., 2013). Identifying central pathogenic factors and intervening upon them can yield benefits for broader biological mechanisms that extend health span. Moreover, these factors may be valuable biomarkers in clinical trials testing geroscience‐based therapies which require intermediate endpoints measured on a shorter timescale than lifespan itself.

Progress identifying the core mechanisms of aging has been hindered by the tradeoff of either measuring large numbers of analytes in fewer people with shorter follow‐up time, or smaller numbers of analytes in more people with longer follow‐up. An important study design that involves measurements of many analytes in a prospective, large, general population cohort followed for a long period of time could minimize selection and survival biases, wherein the long‐lived cases and controls could have been given the same opportunity to achieve longevity and had comparable life experiences from the same birth cohorts (Newman & Murabito, 2013).

Furthermore, all analytes are not equal in their potential utility as clinical biomarkers (Rutledge et al., 2022). Proteins are likely best suited as translational biomarkers because they are often end products of genetics and epigenetics, direct regulators of cellular pathways, and the therapeutic targets of many approved drugs (Moaddel et al., 2021). Proteomic measurements within aging epidemiologic cohorts have been described (Lehallier et al., 2019; Menni et al., 2015; Sathyan, Ayers, Gao, Milman, et al., 2020; Tanaka et al., 2018). However, the majority of existing proteomic studies are of chronologic age, include a narrower set of protein measurements, do not accrue enough follow‐up time to observe large numbers of long‐lived cases, or do not include replication in an independent dataset with similar analytic methods.

The current study attempted to build on prior research with the overall goal of identifying a set of blood‐based biomarkers of human longevity that could be prioritized for translation in geroscience trials. We leveraged data from two large population‐based longitudinal cohorts, the Cardiovascular Health Study (CHS) (Fried et al., 1991) and the Age Gene/Environment Susceptibility‐Reykjavik Study (AGES‐Reykjavik) (Harris et al., 2007), to study the proteomic signature of longevity using the SOMAscan proteomics platform. The two cohorts have been well‐characterized with respect to early old age and have accrued substantially long follow‐up times (median CHS 16 years, AGES‐Reykjavik 11 years) that have allowed a large number of participants the opportunity to live to age 90 or beyond. We performed a primary proteomic analysis of 4985 plasma proteins in CHS and compared two outcomes of longevity (survival to 90 and overall survival). Significant associations were validated in AGES‐Reykjavik. We also developed and validated a protein‐based prediction model for longevity. Furthermore, we examined how measures of physical and cognitive function might mediate the associations between proteins and longevity in CHS, motivated by the theory that factors in blood that promote longevity produce their beneficial effects via improvements in organismal function, as observed from experiments in model systems using heterochronic parabiosis and heterochronic blood transfer (Conboy et al., 2005).

## 2 METHODS

### 2.1 Study population

#### 2.1.1 Cardiovascular Health Study (CHS)

The CHS (Fried et al., 1991) is a prospective cohort study of 5888 community‐dwelling individuals 65 years of age or older from four US communities: Sacramento County, CA; Washington County, MD; Forsyth County, NC; and Allegheny County, PA. The study recruited an initial cohort of 5201 men and women in 1989–1990, and an additional cohort of 687 African Americans in 1992–1993. Participants underwent extensive annual clinical examinations through 1999 which measured traditional cardiovascular risk factors and measures of subclinical disease, as well as phone interviews every 6 months. Deaths were identified by household contacts, consulting obituaries, medical records, death certificates, National Death Index, and Centers for Medicare and Medicaid Services data; 100% follow‐up for ascertainment of mortality status was achieved. Each participant provided written informed consent, and Institutional Review Boards approved the study protocol at each site.

Plasma proteins were measured in 3188 CHS participants who had unthawed samples in 1992–1993 (the third examination for the original cohort and first for the African American cohort). We excluded participants who had flagged (*n* = 3) or unnormalized proteomics data (*n* = 14) or had missingness on covariates (*n* = 104), resulting in an analytic sample of 3067 CHS participants, with a median (IQR) follow‐up of 16 (11–21) years.

#### 2.1.2 Age Gene/Environment Susceptibility‐Reykjavik (AGES‐Reykjavik) study

The AGES‐Reykjavik study is a population‐based study of older men and women in Iceland designed to examine genetic susceptibility and gene/environment interactions in relation to phenotypes of old age (Harris et al., 2007). Initial extensive interviews, examinations, and measurements of 5764 participants, all survivors of the previous Reykjavik Study, were conducted in the years 2002–2006. The multitude of examinations were focused on measures of cardiovascular health and measures related to geriatrics and frailty. Mortality status was ascertained via a mortality registry from the Icelandic Directorate of Health, up to the 31 January 2022. Plasma proteins were measured in samples taken from 5457 participants at the time of their initial examination. Of those, 624 participants were still alive and had not reached the age of 90 at the end of follow‐up and were therefore excluded as it was unknown whether they would meet the primary endpoint. After the further exclusion of 143 participants due to missing covariate data, the number of participants included in the analysis was 4690. These participants had a median (IQR) follow‐up of 11 (7–15) years.

### 2.2 Measurements

Sociodemographic factors, including age, sex, Black race, education, smoking status (current/not current), pack‐years, and alcohol (drinks per week) were determined by self‐report in CHS. Blood pressure was measured twice, and averaged values were used. Anthropomorphic measures included weight (kg), height (cm), and waist (cm). Fasting blood samples were analyzed for glucose, total cholesterol, and high‐density lipoprotein cholesterol. Low‐density lipoprotein cholesterol was calculated according to Friedewald equation. Medication use was determined by a medication/drug inventory (Psaty et al., 1992). Diabetes mellitus was defined as the use of insulin or oral hypoglycemic medications or fasting serum glucose ≥126 mg/dL or non‐fasting serum glucose ≥200 mg/dL. Gait speed (m/s, measured over a 15‐foot (4.6 m) course at normal pace) and grip strength (kg, measured in the dominant hand using Jamar isometric handheld dynamometer) were used as indicators of physical function. The Modified Mini‐Mental State Examination (3MSE) scores (Teng & Chui, 1987) were used as an evaluation of cognitive ability and the digit symbol substitution test (DSST) (Wechsler, 1981) was administered as an indicator of processing speed. For the purposes of this analysis, all CHS measures were obtained in the 1992–1993 examination. In AGES‐Reykjavik, sociodemographic factors such as education, smoking status (current/former/never), pack‐years, alcohol use, and medication use were derived from questionnaires. For the 63 (1%) participants that reported cigarette smoking but no amount, pack‐years of smoking were imputed by kNN‐imputing using other covariates. Participants had their height, weight, abdominal circumference, and blood pressure measured, and fasting blood samples analyzed as previously described, with the 3MSE and the DSST administered to all participants (Harris et al., 2007). Gait speed was measured during a 6‐meter‐long walk at participants' usual pace and grip strength was measured by a computerized dynamometer in the dominant hand (Siggeirsdottir et al., 2007).

### 2.3 Proteomics

The SOMAscan 5k platform was used to measure 4985 plasma proteins (annotated to 4172 protein analytes) in 2020 for CHS (Version 4.0) (Austin et al., 2022). Likewise, 4783 SOMAmers measuring human proteins (annotated to 4137 protein analytes) were used for proteomic analysis of the serum of 5457 AGES‐Reykjavik participants. The SOMAscan assays (SOMALogic, Boulder, CO) have been described previously (Mehan et al., 2013), and the clinical and analytic validity of the aptamer‐based assay method has been demonstrated in recent reports, including in AGES‐Reykjavik (Emilsson et al., 2018; Ngo et al., 2016). The differences in sample type are considered minimal as the SOMAscan platform provides relative quantification of protein and all measurements are standardized.

A total of 3924 proteins (79% of CHS proteins) were measured in both CHS and AGES‐Reykjavik and a complete list of these proteins is included in Table S1. Adaptive normalization by maximum likelihood (ANML) was performed in CHS in order to remove sample or assay biases. The median intra‐assay (inter‐assay) coefficient of variation was 3.4% (4.4%) using quality control samples and intraclass correlation coefficient was 0.66 in 100 samples from two examination cycles for CHS 5 years apart (Austin et al., 2022; Ngo et al., 2016). CHS protein data were log2‐transformed and standardized (mean = 0, SD = 1) for this analysis. As previously described, AGES‐Reykjavik protein data were transformed with a Box‐Cox transformation, providing a standardization with a mean of around 0 and a standard deviations (SD) of 1, and extreme outliers (0.2% of values) were excluded from analysis with values regarded as missing in analyses (Gudmundsdottir et al., 2020).

### 2.4 Outcomes

The primary outcome was survival to 90 or older (yes or no). Exceptional longevity phenotypes (e.g., nonagenarians) have been frequently used as omnibus aging outcomes (Newman & Murabito, 2013). The cutoff of 90 years was chosen as approximately three SD above the mean age in CHS participants (mean [SD] age = 74.4 [4.9]). Moreover, age 90 also represents a well‐established marker of exceptional longevity in line with previous studies (Häberle et al., 2021; Odden et al., 2019; Zabielska et al., 2019), and using this cutoff could facilitate an easier comparison between studies. The secondary outcome was overall survival defined by time to death.

### 2.5 Statistical analysis

#### 2.5.1 Individual protein analysis

Logistic regression was used to estimate the odds ratios (ORs) of individual plasma protein levels (log‐transformed and standardized) with survival to 90. Cox regression was used to estimate the hazard ratios (HRs) of protein levels with overall survival (time to death). Models were adjusted for age, sex, race (in CHS only as all AGES participants were of the same race), height, weight, smoking status, and pack‐years of smoking. Models in CHS were also adjusted for waist circumference, while replication analyses in AGES‐Reykjavik were adjusted for abdominal circumference. Multiple testing was corrected by the Bonferroni method using the number of principal components explaining 99% of the total variability in proteins (*n* = 2505), corresponding to a significance threshold of 0.05/2505 = 2.0E−5. This approach has been used previously (Liu et al., 2022); it identifies the number of truly independent proteins (independent tests) to mitigate the false positive while avoiding overlooking true positives. The same significance threshold was used for the replication analyses in AGES‐Reykjavik. A Venn diagram was used to visualize proteins that were statistically significantly associated with both outcomes. The proteins that were significantly associated with survival to 90 were included in the subsequent analysis (*n* = 211). Additionally, we performed several sensitivity analyses using data from the exploratory cohort (CHS): (1) we assessed the associations of each protein with chronological age using linear regression adjusted for the same covariates and compared the results with the other two models; (2) we tested the sex‐protein interaction and performed a sex‐stratified analysis to capture potential sex‐different proteomics of longevity.

#### 2.5.2 Prognostic analysis

A least absolute shrinkage and selection operator (LASSO) logistic regression was performed to identify the most robust prognostic proteins and establish an optimal model to predict longevity using the “glmnet” R package and CHS data. Ten‐fold cross‐validation was performed based on mean squared error criterion (MSE) and used to select the penalty regularization parameter lambda. The largest lambda, at which the MSE is within one standard error of the smallest MSE (lambda.1se), was fixed as the penalty parameter to prevent an overfitting effect. The variables included in the LASSO selection were basic demographic and traditional predictors (age, sex, race, height, weight, waist, smoking status, and pack‐years) with no penalization and protein measures (*n* = 211) with equal penalization. We trained the model parameters using 50% randomly selected CHS individuals (training set: *n* = 1533) and tested the performance of the remaining 50% CHS individuals (test set: *n* = 1534). The relative importance (i.e., magnitude of the beta coefficients, because the protein levels were standardized) of the selected proteins was presented using a heatmap where an extreme color indicated a greater importance. Area under the ROC (receiver operating characteristic) Curve (AUC) was used to compare the classification of the basic model (predictors including age, sex, race, height, weight, waist, smoking status, and pack‐years) and protein model (basic model plus protein predictors identified by LASSO regression) in the test set. Subsequently, we examined the model performance using the whole AGES‐Reykjavik cohort as a validation cohort, using the variables selected for the LASSO model that were available in the AGES‐Reykjavik data. DeLong's test was used to compare the AUCs between two prognostic models.

#### 2.5.3 Statistical mediation analysis

Casual mediation analysis was performed to examine whether the associations between proteins and longevity were mediated by functional measures using the “mediate” R package. Mediation analysis was only performed in CHS because the mediation effect is more prone to variability due to underlying population characteristics, and therefore comparability between studies is more dependent on differences in population than the preceding association analyses (Xue et al., 2022). Potential mediators for the protein‐longevity associations included gait speed, grip strength, DSST, and 3MSE. The mediator model was fitted using linear regression to assess the associations between proteins and each mediator with adjustment for age, sex, race, height, weight, waist, smoking status, and pack‐years. The outcome model was fitted using logistic regression to assess the associations between proteins and longevity with adjustment for the same covariates set. An interaction term of protein*mediator was included if the *p* value for interaction was <0.1. Confidence intervals were estimated by quasi‐Bayesian approximation with 1000 Monte Carlo draws. Average causal mediation effect (ACME) was presented and interpreted as the expected difference in the potential outcome when the mediator took the value that would realize under the treatment condition as opposed to the control condition (one standard deviation increase in the log‐transformed protein value in this case), while the treatment status itself is held constant. Multiple testing was corrected by Bonferroni method to adjust the number of proteins and mediators tested (*p* < 0.05/[211*4] = 6.6E−5) and the Venn diagram was used to show the differences in the number of proteins that are statistically mediated among four mediators.

## 3 RESULTS

### 3.1 Baseline characteristics

Of the 3067 CHS participants included, 1363 survived to age 90 years or older. Women, non‐current smokers, those with lower weights, lower waist circumference, and less smoking pack‐years were more likely to survive to 90 or older. Additionally, those who reached 90 or older had higher HDL levels and were less likely to have diabetes and use antihypertensive medications. Of the 4690 AGES‐Reykjavik participants included, 2172 survived to 90 years of age. Similar demographic differences were observed among those that reached 90 years as in CHS, with the addition that survivors to 90 years were less likely to be educated, shorter, had higher systolic blood pressure and lipoprotein levels, and were more often on cholesterol‐lowering medication, with no difference in usage of antihypertensives (Table 1).

**TABLE 1:** Baseline characteristics of CHS and AGES‐Reykjavik participants.

Variables	CHS: Survival to 90			AGES: Survival to 90		
	No (*n* = 1704)	Yes (*n* = 1363)	*p* value	No (*n* = 2518)	Yes (*n* = 2172)	*p* value
Age, years	73.5 (4.1)	75.6 (5.5)	<0.001	75.4 (4.7)	79.7 (4.9)	<0.001
Sex						
Female	961 (56%)	907 (67%)	<0.001	1258 (50%)	1350 (62%)	<0.001
Male	743 (44%)	456 (33%)		1260 (50%)	822 (38%)	
Race				–	–	–
Black	288 (17%)	204 (15%)	0.160			
Not Black	1416 (83%)	1159 (85%)				
Clinic				–	–	–
Bowman Gray	440 (26%)	334 (26%)	0.140			
Davis	415 (24%)	376 (28%)				
Hopkins	386 (23%)	278 (20%)				
Pittsburgh	463 (27%)	375 (28%)				
Education						
Less than high school	223 (13%)	163 (12%)	0.380	582 (23%)	577 (27%)	0.007
High school or above	1481 (84%)	1200 (88%)		1930 (77%)	1592 (73%)	
Height, cm	165.6 (9.7)	163.7 (9.0)	<0.001	168.4 (9.4)	165.4 (9.2)	<0.001
Weight, kg	72.7 (14.8)	70.9 (13.3)	<0.001	77.2 (15.6)	73.4 (13.2)	<0.001
Waist, cm^a^	97.2 (13.3)	96.1 (12.3)	0.018	101.4 (12.7)	100.0 (11.2)	<0.001
Current smoker^b^						
Not current	1489 (87%)	1301 (96%)	<0.001	2094 (83%)	2011 (93%)	<0.001
Current	215 (13%)	62 (4%)		424 (17%)	161 (7%)	
Pack‐years^c^	20.8 (27.6)	10.2 (18.1)	<0.001	15.8 (21.0)	8.9 (15.4)	<0.001
Current drinker	774 (46%)	654 (48%)	0.171	1609 (64%)	1339 (62%)	0.107
Systolic blood pressure, mmHg	136.3 (21.5)	134.9 (20.9)	0.081	141.9 (21.0)	144.1 (21.0)	<0.001
Diastolic blood pressure, mmHg	71.5 (11.2)	70.8 (10.9)	0.069	74.3 (9.8)	72.9 (9.6)	<0.001
Low‐density lipoprotein, mg/dL	127.2 (34.2)	129.3 (32.7)	0.094	131.1 (40.5)	138.1 (40.0)	<0.001
High‐density lipoprotein, mg/dL	52.7 (14.5)	55.3 (14.0)	<0.001	59.7 (17.4)	63.1 (17.0)	<0.001
Grip strength, kg^d^	28.7 (10.1)	27 (9.4)	<0.001	306 (113.0)	281 (107.0)	<0.001
Gait speed, m/s	0.9 (0.2)	0.9 (0.2)	0.013	0.9 (0.2)	0.9 (0.2)	0.603
DSST, score	41.9 (13.4)	40.5 (13.5)	<0.001	27.7 (11.0)	27.0 (10.5)	0.020
3MSE, score^e^	91.7 (7.9)	91.4 (8.0)	0.067	26.4 (3.3)	26.4 (3.0)	0.93
Diabetes	303 (18%)	111 (8%)	<0.001	383 (15%)	208 (10%)	<0.001
Hypertension medication	886 (52%)	571 (42%)	<0.001	1631 (65%)	1423 (66%)	0.616
Lipid lowering medication	128 (8%)	108 (8%)	0.488	678 (27%)	430 (20%)	<0.001

*Note*: Data were mean (standard deviation) or *n* (%). In AGES‐Reykjavik, participants whose data are shown had missing data on gait speed (98), grip strength (365), DSST (127), educational attainment (9), lipoprotein levels (4), and blood pressure (1).

Abbreviations: 3MSE, Modified Mini‐Mental State Examination; CHS, Cardiovascular Health Study; DSST, digit symbol substitution test.

^a^ Abdominal circumference, not waist circumference, was measured in AGES‐Reykjavik.

^b^ Analyzed as ever/former/current smoker in AGES‐Reykjavik.

^c^ Number of pack‐years was imputed for 63 participants reporting cigarette smoking but no pack‐years.

^d^ The unit of measurement in AGES‐Reykjavik is Newton.

^e^ Shown are results from the Mini‐Mental State Examination in AGES‐Reykjavik.

### 3.2 Proteins associated with longevity

**FIGURE 1:**
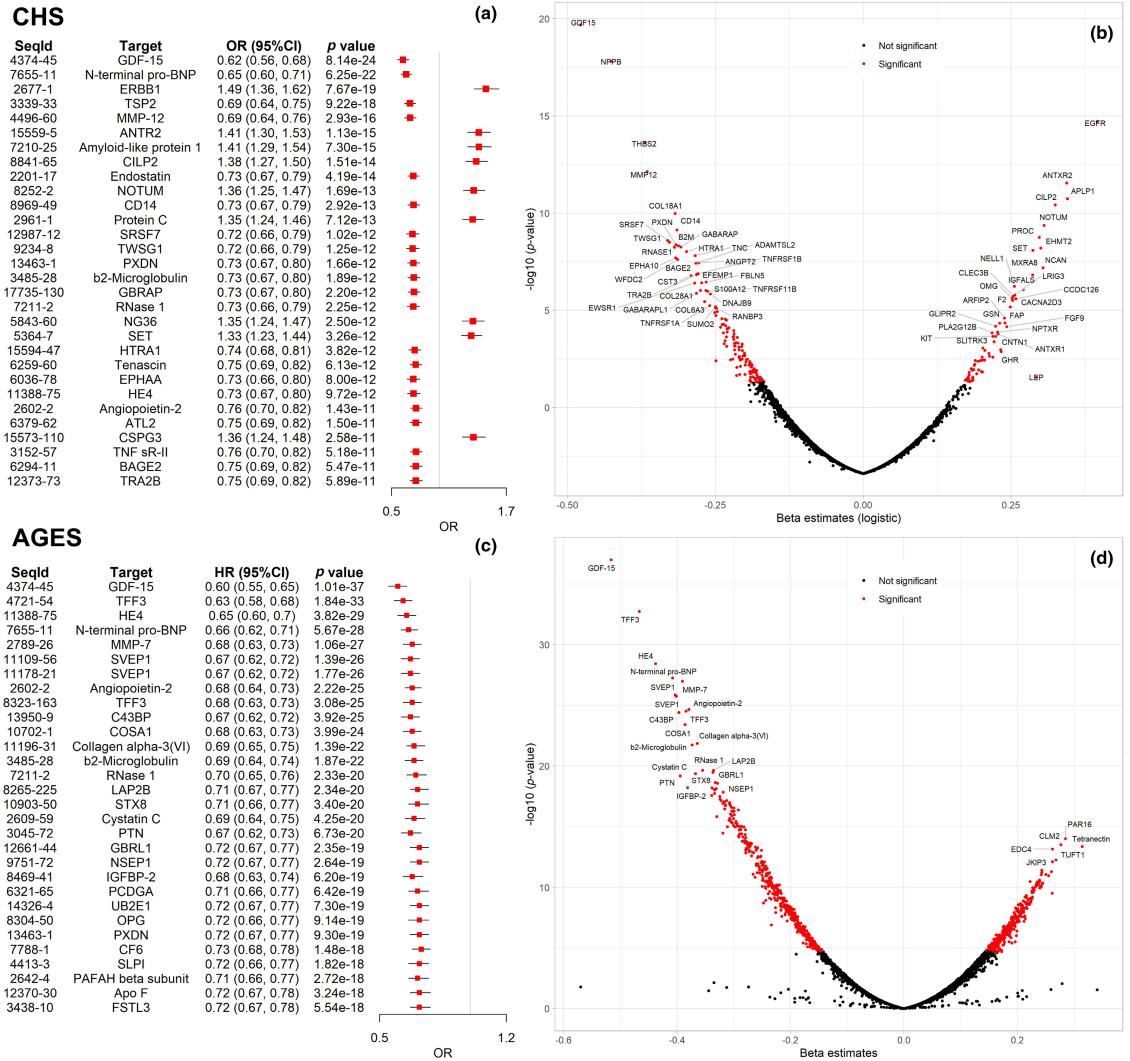
Logistic regression of proteins associated with survival to 90. (a) Odds ratios of top 30 significant proteins associated with survival to 90. (b) Volcano plot summarizing associations of all proteins (*n* = 4985) with survival to 90 in CHS. (c) Odds ratios of top 30 significant proteins associated with survival to 90 in AGES‐Reykjavik. (d) Volcano plot summarizing associations of all proteins (*n* = 4783) with survival to 90 in AGES‐Reykjavik.

Logistic regression identified 211 plasma proteins that were statistically significantly associated with survival to 90 (longevity) in CHS. Of these, 142 proteins were associated with lower odds of survival to 90 (*β* < 0). The strongest associations were for GDF‐15 (OR [95%CI] per standard deviation log2 protein = 0.62 [0.56, 0.68], unadjusted *p* value = 8.14E−24), N‐terminal pro‐BNP (0.65 [0.60, 0.71], 6.25E−22), and ERBB1 (1.49 [1.36, 1.62], 7.67E−19). In AGES‐Reykjavik, 766 proteins were associated with survival to 90, including 439 proteins associated with lower odds. The top 30 proteins with the strongest associations with survival to 90 in CHS and AGES are shown in Figure 1. Of the 211 associated proteins in CHS, data were available for 168 (80%) in AGES‐Reykjavik. Of these, 105 (63%) were associated with survival to 90 after adjustment for multiple testing, and 137 (82%) were nominally associated (unadjusted *p* < 0.05). For these 168 proteins, the directionality of effect was consistent between cohorts for 155 (92%). The associations of GDF‐15 (0.60 [0.55, 0.65], 1.01E−37) and N‐terminal pro‐BNP (0.66 [0.62, 0.71], 5.67E−28) with survival to 90 were also among the strongest in AGES‐Reykjavik, while the association of ERBB1 (1.10 [1.03, 1.17], 0.007) was not significant after multiple testing adjustment. Other proteins among the top 30 in both cohorts included b2‐microglobulin, RNase 1, HE4, angiopoietin‐2, and PXDN. TFF3 (0.63 [0.58, 0.68], 1.84E−33) had a strong association in AGES‐Reykjavik while it was marginal in CHS (rank of *p* value = 218). The odds ratios of these 168 replicated proteins with survival to 90 in CHS and AGES‐Reykjavik were shown in Table S2. The complete results for 5k proteins in CHS are provided in Table S3.

### 3.3 Proteins associated with overall survival

**FIGURE 2:**
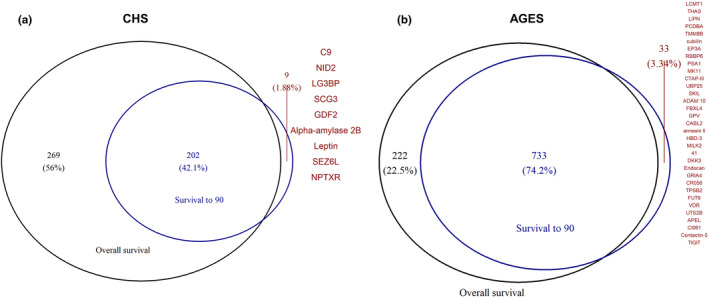
Venn diagram showing the difference of the significant proteins for survival to 90 and overall survival in CHS (a) and AGES‐Reykjavik (b). Labeled proteins are those significantly associated with survival to 90 but not with overall survival.

In total, 471 proteins were statistically significantly associated with overall survival in CHS, and 363 of these were associated with a higher risk of death (Figure S1). The strongest associations were for GDF‐15 (HR [95%CI] = 1.39 [1.33, 1.46], unadjusted *p* value = 4.12E−43) and N‐terminal pro‐BNP (1.36 [1.30, 1.42], 2.26E−42). Among all proteins analyzed in AGES‐Reykjavik, 955 proteins were associated with overall survival wherein 539 were associated with an increased death risk, and the most significant associations were also for GDF‐15 (1.37 [1.23, 1.42], 2.41E−61) and N‐terminal pro‐BNP (1.30 [1.25, 1.34], 3.18E−49). Of the 211 proteins associated with longevity in CHS, 202 were also statistically significantly associated with overall survival (Figure 2a). In AGES‐Reykjavik, 733 of the 766 proteins associated with longevity were also associated with overall survival (Figure 2b). These proteins are associated with both outcomes in the same direction. We found 9 proteins (C9, NID2, LG3BP, SCG3, GDF2, Alpha‐amylase 2B, Leptin, SEZ6L, and NPTXR) in CHS and 33 proteins in AGES that were associated with longevity but not significantly associated with overall survival, although the corresponding *p* values were marginal and the directionalities of effect were consistent between two outcomes (Table S4). Sensitivity analyses showed that the proteomics profile linked to longevity includes a distinct subset of 78 proteins that do not correlate with chronological age (Figure S2). We found no significant sex‐protein interaction (Bonferroni *p* value for interaction >0.05) and the protein signature associated with longevity appeared to be consistent across sex (Figure S3).

### 3.4 Prognostic model for longevity

**FIGURE 3:**
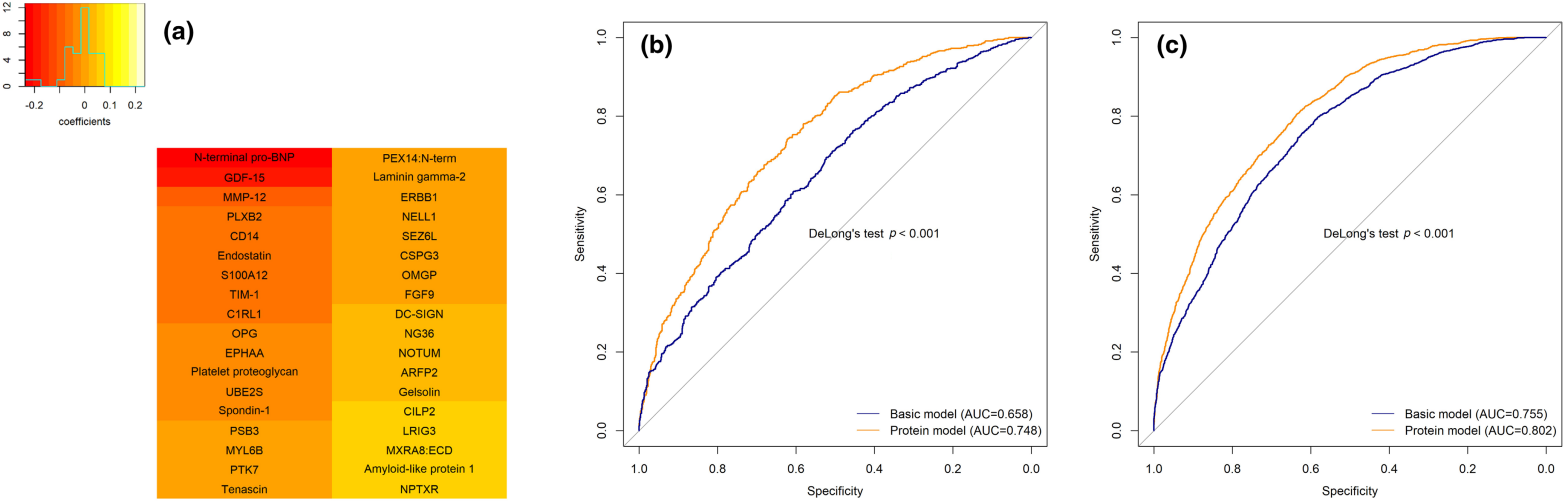
Prognostic analysis of proteins for survival to 90 (a) Proteins chosen by LASSO with 10‐fold cross‐validation using within one standard error of the minimum criteria (lambda.1se). (b) ROC curves verified the prognostic performance of the proteins chosen by LASSO in CHS. (c) ROC curves validated the prognostic performance of the proteins model in AGES‐Reykjavik. Proteins are colored according to the magnitude of the coefficients. The basic model included age, sex, race (in CHS only), height, weight, waist circumstances in CHS or abdominal circumstances in AGES‐Reykjavik, smoking status, and pack‐years. The protein model was the basic model plus 36 proteins in (a) and 27 out of 36 proteins were available and used in the AGES‐Reykjavik model.

The LASSO logistic regression analysis selected 36 prognostically relevant proteins for predicting longevity using CHS data (Figure 3a). Of these, 20 proteins had a negative coefficient, indicating a higher concentration was associated with a lower likelihood of achieving longevity. The proteins with the greatest prognostic value were N‐terminal pro‐BNP, GDF‐15, and MMP‐12, all of which were negatively associated with longevity (*β* < 0). The proteins NPTXR and Amyloid‐like protein 1 were selected by LASSO and had a relatively high positive association with longevity (*β* < 0). The basic model with traditional predictors generated an AUC of 0.658 and the protein model increased the AUC to 0.748 (Figure 3b) with a DeLong's test showing a significant difference (*p* value < 0.001). Of the 36 proteins selected in CHS, data were available for 27 in AGES‐Reykjavik. A logistic prognostic model for longevity using these proteins plus covariates was trained and performed well in AGES‐Reykjavik, with an AUC of 0.802 compared with 0.755 for a basic model with traditional covariates only (DeLong's *p* value < 0.001). The ROC curves are shown in Figure 3c. A full list of LASSO‐selected proteins and corresponding coefficients are shown in Table S5.

### 3.5 Mediation analysis

**FIGURE 4:**
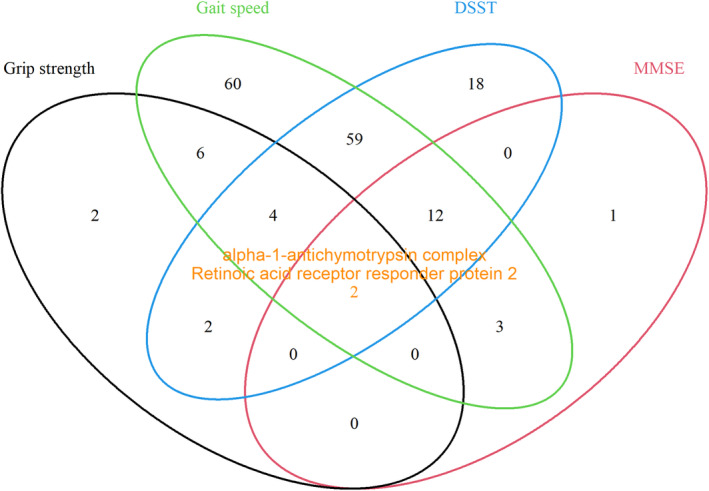
Venn diagram showing protein associations mediated (partially) by four functional measurements including grip strength, gait speed, digit symbol substitution test (DSST), and modified mini‐mental status examination (3MSE) in CHS. A total of 211 proteins that are significantly associated with survival to 90 by logistic regression were included in this analysis and 169 of them were mediated by at least one mediator (2 mediated by 4, 16 mediated by 3, 70 mediated by 2, 81 mediated by 1). The mediating model was fitted using linear regression with each of the four mediators as outcome, and protein levels as exposures and adjusted for age, sex, race, height, weight, waist, smoking status, and pack‐years. The outcome model was fitted using logistic regression with survival to 90 as outcome, protein levels, and each of the four mediators as exposures, and adjusted for the same covariates set. An interaction term of protein*mediators was included if the *p* value for interaction <0.1.

**FIGURE 5:**
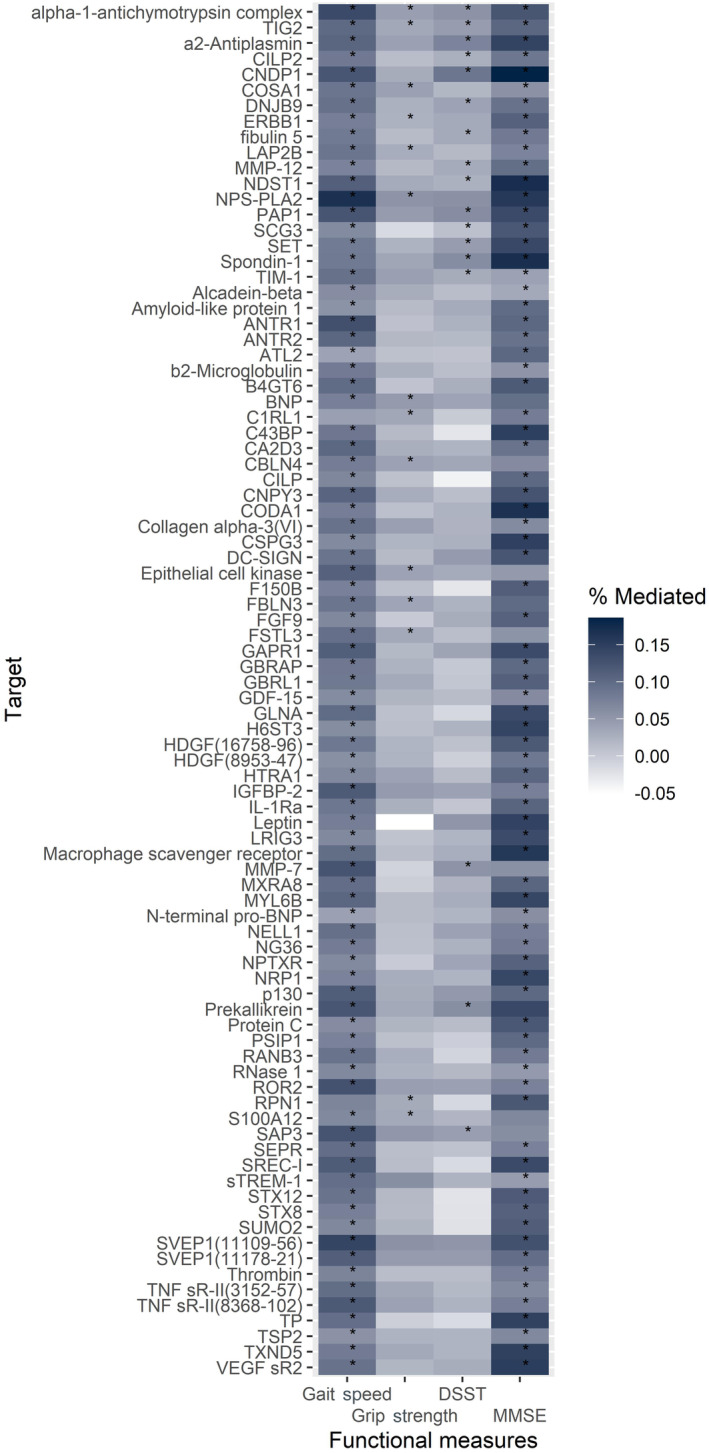
Heatmap depicting the percentage mediated of each functional measurement on the protein‐longevity associations (*n* = 88 mediated by 2+ mediators). Statistically significant mediation is annotated by asteroid *. Repeated targets are distinguished by SeqId.

Causal mediation analysis was performed in CHS and revealed that the associations of 169 proteins (out of 211) with longevity were mediated (partially) by at least one functional measurement (146 by gait speed, 16 by grip strength, 97 by DSST, 18 by 3MSE). Of these, 88 protein associations were mediated by 2 functional measurements and 16 protein associations were mediated by 3 functional measurements. Two protein associations (alpha‐1‐antichymotrypsin‐complex, and retinoic acid receptor responder protein 2) were partially mediated by all four functional measurements (Figure 4). Table S6 shows the proteins whose associations with longevity were statistically significantly mediated by at least two functional measurements (*n* = 88) and the ACME and Figure 5 shows the percentage mediated by four mediators for these associations. The direction of the mediation was consistent across mediators for most proteins. Interestingly, of the 88 protein‐longevity associations mediated by at least 2 functional measurements, there were 77 overlapping between mediation by gait speed and DSST.

## 4 DISCUSSION

In this proteomics study of two large prospective cohorts, we identified plasma proteins significantly associated with longevity in older adults and showed that proteomic profiles can add to traditional predictors to better predict longevity, even over long follow‐up times. The strongest associations in CHS that were replicated in AGES‐Reykjavik were for GDF‐15, NT‐pro‐BNP, b2‐microglobulin, RNase 1, and HE4, providing confidence in such previously identified proteins in aging research. Less‐established markers of mortality in the general population, such as angiopoietin‐2, and PXDN, also had support in both cohorts. Our study design leveraging a longevity outcome, as opposed to overall survival only, paired with long follow‐up time revealed that nearly half (269 out of 471) of proteins associated with overall survival were not associated with exceptional longevity in the CHS, though the strongest associations remained consistent between the two outcomes. A larger share of significant proteins was associated with both overall survival and longevity in AGES‐Reykjavik, which may have occurred due to increased power to detect significant associations in AGES‐Reykjavik. This observation suggests that extrapolating findings from associations with overall survival to longevity might be inappropriate. Moreover, we demonstrate for the first time in proteomics studies of longevity that physical and cognitive function may partially mediate associations between proteins and longevity, and that the amount of mediation may depend in part on which particular functional measures are used in the analysis. Collectively, our analyses highlight that study designs combining broader unbiased proteomics with cohorts of large general population samples and long follow‐up can yield new results on proteomic signature of longevity and aging outcomes may share common underlying mechanisms. These results help identify the most promising (true positive) associations to advance proteins for biomarker candidacy in experimental studies in model systems and, perhaps, in geroscience‐focused human clinical trials.

Previous proteomics research on aging primarily focused on chronological age as the primary outcome, whereas we discovered indications that longevity may be associated with a unique proteomic profile. Nevertheless, a few important SomaScan studies in INTERVAL (Lehallier et al., 2019), TwinsUK (Menni et al., 2015), Baltimore Longitudinal Study of Aging (Tanaka et al., 2018), and LonGenity (Lehallier et al., 2019; Sathyan, Ayers, Gao, Weiss, et al., 2020) have repeatedly identified associations with several proteins, including GDF‐15, N‐terminal pro‐BNP, β2‐microglobulin, and FSTL3, and these proteins were confirmed in the present study to be associated with longevity and survival in older adults from two independent cohorts. Specifically, GDF‐15 has been related to physical decline, diabetes, cardiovascular disease, and mortality (Justice et al., 2018; Liu et al., 2022); N‐terminal pro‐BNP is a known risk indicator for heart failure and coronary artery disease (Kragelund et al., 2005); β2‐microglobulin is identified as a circulating factor that negatively regulates cognitive and regenerative function (Smith et al., 2015). In CHS we also observed an association between the up‐regulated ERBB1 and longer survival, and this was concordant with findings from INTERVAL (Lehallier et al., 2019), LonGenity (Sathyan, Ayers, Gao, Milman, et al., 2020), and ARIC cohorts (Tin et al., 2019), although this protein was only nominally significant in AGES. Additionally, our findings are aligned with MrOs cohort to show that LG3BP and NRP1 are associated with a lower probability of achieving longevity (Orwoll et al., 2020). We also identified other age‐associated proteins, including Rnase 1 (an endonuclease that catalyzes the cleavage of RNA), HE4 (a broad‐range protease inhibitor), and PXDN (a heme‐containing peroxidase that is secreted into the extracellular matrix), among the 30 most significantly associated with longevity. Notably, Rnase 1 was associated with a higher frailty phenotype in a recent SomaScan study of the LonGenity cohort (Sathyan, Ayers, Gao, Weiss, et al., 2020), and the mediation analysis in this study showed that its association with longevity was partially mediated by gait speed and grip strength. Angiopoietin 2 measured in cerebrospinal fluid was previously associated with aging (Baird et al., 2012), consistent with our results using plasma. Angiopoietin 2 is implicated in endothelial dysfunction and atherosclerosis and is associated with mortality in patients with chronic kidney disease (David et al., 2012). Apart from the above proteins, an important protein predictor for longevity was MMP‐12 (Lehallier et al., 2019; Menni et al., 2015), which has been strongly implicated in the aging process in several tissues, such as skin and brain (Liu et al., 2013), and the development of age‐related diseases, such as neurodegenerative disorders (Freitas‐Rodríguez et al., 2017). A few longevity proteins (TNF sR‐II and TNF sR‐I) were also previously reported to be significantly reduced by aging interventions such as calorie restriction (Aversa et al., 2023). Based on the “SASP Atlas” (Basisty et al., 2020), a comprehensive and curated proteome‐based databased of senescence‐associated secretory phenotypes, many of the longevity‐associated proteins (*n* = 14) are considered SASP, and four (cystatin C, GDF‐15, IGFBP‐2, and HSP70 protein 8) are considered “core” SASP—proteins that are consistently stimulated by a variety of senescent stimuli. Further investigation of these proteins is warranted to determine whether these associations are causal or simply correlative.

Proteomic data have been used to predict chronological age and develop proteomic clocks (Lehallier et al., 2020; Sathyan, Ayers, Gao, Weiss, et al., 2020; Tanaka et al., 2018). In a prior SomaScan study, proteomic clocks were built to predict chronological age using a composite score of proteins selected by machine learning techniques. The predicted age was well correlated with observed chronological age (correlation range from 0.8 to 0.98). While a worthy goal in and of itself, correlation with chronologic age is not a surrogate for predicting incident events, which is traditionally more difficult, but also a vital benchmark to pass in order to advance biomarkers for use in clinical trials. In this study, we employed LASSO for feature selection and built a parsimonious protein‐based prediction model for survival to 90 years of age over a long period (median 16 years). We found that the addition of selected protein features significantly improved the classification performance compared to a model with only traditional risk factors. The established model had a robust performance in the replication cohort, although we observed a smaller increase in performance above the basic model than was seen in the training/testing samples. This finding might be explained by the different demographics of the two cohorts (e.g., the older age at the start of follow‐up in AGES‐Reykjavik) and that fewer proteins were available in the replication cohort. We also observed that when all the proteins were considered for selection in the LASSO models, many fewer proteins were ultimately selected in the final prediction model (fewer than 40) compared with the number of significant associations between individual proteins and longevity (hundreds) derived from models testing the association of each protein with longevity. Operationally, because our prediction model distilled a minimal set of proteins from a large unbiased sample of potential predictors, future observational and interventional studies hoping to measure biomarkers for longevity prediction could substantially reduce their assay costs by focusing on these biomarkers, rather than performing additional unbiased testing.

Previous studies have associated more limited sets of circulating biomarkers with functional changes in older adults (Newman et al., 2016; Peterson et al., 2016), and demonstrated that functional status significantly modified the mortality risk in older adults (Odden et al., 2012; Peralta et al., 2014). Evidence for mediation of the proteome through physical and cognitive function on longevity would support the theory that there are common underlying mechanisms for aging outcomes. We tested four functional measurements as potential mediators and found that 169 (169/211) protein‐longevity associations were partially mediated by at least one of them. Although correlative only, this observation is consistent with the theory that diverse circulating proteins may exert their effects on longevity through unified, higher‐order functional changes and that functional measurements may serve as valid intermediate endpoints for interventions attempting to modulate proteins to affect longevity. Indeed, recent animal experiments suggest that longevity protein klotho activates platelet factors to mediate cognition enhancement in aging mice (Park et al., 2023). Moreover, findings from heterochronic parabiosis and heterochronic blood exchange experiments have demonstrated that young blood or plasma may have pro‐regenerative factors and exposure to young blood could rejuvenate physiological function and multiple tissues in aged animals (Rebo et al., 2016; Villeda et al., 2014), but identifying the causal circulating factors and how they exert their effects is still challenging. Our data provide insight on which circulating proteins may be the most fruitful for additional causal studies, and how they may exert their effects, that is, through physical and cognitive function or not.

Notably, the manifestation and magnitude of the mediation effects vary drastically depending on which functional measure is being evaluated. Similar to the non‐synonymous protein associations between overall and exceptional survival, this observation supports the notion that using phenotypes even within the same domain (physical function or cognitive function) can have markedly different effects on results. Measuring high‐dimensional data, such as any omics type, does not guard against the fact that the omics associations remain driven by which phenotype they are linked with. In our analysis, gait speed significantly mediated (partially) the largest number of protein‐longevity associations, followed by DSST, 3MSE, and grip strength. Gait speed and DSST may partially mediate more protein‐longevity associations due to the physiology they reflect, and they seem to exhibit more substantial mediation effects (a larger percentage mediated) compared to the other two measures. Specifically, gait speed is considered a higher‐order function that collectively captures a larger proportion of human body physiology than grip strength (Ferrucci et al., 2000; Stessman et al., 2017), and DSST measures general and unspecific processing speed and is more sensitive to slight changes in higher‐level cognition than 3MSE, which has a ceiling effect (Proust‐Lima et al., 2007). Moreover, DSST likely shares more common inputs with gait speed than it does with grip strength (Chou et al., 2019). Consistent with this study's results, our previous investigation of proteomic associations with the longitudinal decline of gait speed versus grip strength demonstrated unique protein profiles associated with each functional change (Liu et al., 2022).

The strengths of our study include using data from two well‐characterized community and population‐based longitudinal cohorts with long follow‐up times paired with the largest panel of plasma proteins measured in a study of human longevity. Our study also had several limitations. The SOMAscan aptamers did not perfectly overlap between the two cohorts and thus we were not able to replicate the entire protein set. The SOMAscan provides relative but not absolute quantification of protein level, which precludes direct comparisons with results derived by other protein measurement methods. This limitation, however, is balanced by the sensitivity, specificity, repeatability, and availability of the SOMAscan aptamers that allow accurate and comparable measurement across studies. Pre‐processing and standardization of proteomic measures were conducted based on slightly different protocols between the two cohorts, while the relative level between proteins would still maintain despite different standardization procedures. Although many aptamers were validated with orthogonal measurement (mass spectrometry) during SOMAscan development, not all aptamers have been orthogonally validated. Although proteins are typically gene‐product effectors and more chemically stable biomarkers, products of transcription and metabolism may also be valuable predictors of longevity. CHS participants with unthawed plasma samples may exhibit different biology than those without and excluding AGES‐Reykjavik participants who would not have had the chance to reach 90 years of age during the follow‐up period made the sample no longer represent the full AGES cohort. Large aging proteomic studies conducted with different technologies are needed to provide a comprehensive picture of the aging proteome in addition to validating our findings in generalized populations and diverse sex and race subgroups.

In conclusion, we identified a common set of proteins associated with and predicting survival to age 90 derived from a large unbiased sampling of proteins measured in two large epidemiologic cohorts. Furthermore, we show how these protein‐longevity associations vary depending on the survival phenotype used to define them, and how they are differentially mediated by multiple functional measurements. These proteins can serve as a validated starting set for further study of their causal or correlative importance, particularly as potential intermediate endpoints in interventional studies.

## AUTHOR CONTRIBUTIONS

X.L., J.L.S., and M.C.O. configured the concept and design of study and manuscript preparation. TRA contributed to the acquisition of the data. XL analyzed the CHS data. GTA performed the replication analysis in AGES‐Reykjavik. All authors contributed to the critical revisions of the manuscript.

## CONFLICT OF INTEREST STATEMENT

BMP serves on the Steering Committee of the Yale Open Data Access Project funded by Johnson & Johnson. JLS is an employee of Vertex Pharmaceuticals at the time of publication. LLJ is an employee and stockholder of Novartis.

## Supporting information


**TABLE S1.** List of proteins assayed on SOMAscan™ platform in CHS and AGES‐Reykjavik.
**TABLE S2.** Replication of 168 significant proteins associated with survival to 90 in CHS and AGES‐Reykjavik.
**TABLE S3.** Complete results of proteins (*n* = 4985) associated with survival to 90 in CHS.
**TABLE S4.** Difference of the significant proteins for survival to 90 and overall survival in CHS and AGES‐Reykjavik.
**TABLE S5.** Coefficients of the proteins chosen by LASSO.
**TABLE S6.** Average causal mediation effect (ACME) of 4 functional measurements for partially mediating the associations of proteins with survival to 90.
**FIGURE S1.** Survival analysis of proteins associated with overall survival by Cox model. (a) Hazard ratios of top 30 significant proteins associated with overall survival in CHS. (b) Volcano plot summarizing the associations of all proteins (*n* = 4985) with overall survival in CHS. (c) Hazard ratios of top 30 significant proteins associated with overall survival in AGES‐Reykjavik. (d) Volcano plot summarizing the associations of all proteins (*n* = 4783) with overall survival in AGES‐Reykjavik.
**FIGURE S2.** Venn diagram showing the difference of the significant proteins for chronological age, survival to 90, and overall survival in CHS.
**FIGURE S3.** Sex‐specific associations of top 50 proteins with survival to 90 in CHS.

Table S1.

Table S3.

## Data Availability

The data are available from the Cardiovascular Health Study and Age Gene/Environment Susceptibility‐Reykjavik Study in accordance with the policies and procedures of the study; please contact the corresponding author for details. Analytic R codes are available at https://github.com/XiaojuanLiu/Protein‐Longevity‐Analysis.
