# Supplementary material for: Plasma proteomic signature of human longevity
Source: Aging Cell. 2024 Mar 5;23(6):e14136. doi: 10.1111/acel.14136 (PMC11166369; doi:10.1111/acel.14136)
Supplement: Supplementary file 1 — TABLE S1. List of proteins assayed on SOMAscan™ platform in CHS and AGES‐Reykjavik. TABLE S2. Replication of 168 significant proteins associated with survival to 90 in CHS and AGES‐Reykjavik. TABLE S3. Complete results of proteins (n = 4985) associated with survival to 90 in CHS. TABLE S4. Difference of the significant proteins for survival to 90 and overall survival in CHS and AGES‐Reykjavik. TABLE S5. Coefficients of the proteins chosen by LASSO. TABLE S6. Average causal mediation effect (ACME) of 4 functional measurements for partially mediating the associations of proteins with survival to 90. FIGURE S1. Survival analysis of proteins associated with overall survival by Cox model. (a) Hazard ratios of top 30 significant proteins associated with overall survival in CHS. (b) Volcano plot summarizing the associations of all proteins (n = 4985) with overall survival in CHS. (c) Hazard ratios of top 30 significant proteins associated with overall survival in AGES‐Reykjavik. (d) Volcano plot summarizing the associations of all proteins (n = 4783) with overall survival in AGES‐Reykjavik. FIGURE S2. Venn diagram showing the difference of the significant proteins for chronological age, survival to 90, and overall survival in CHS. FIGURE S3. Sex‐specific associations of top 50 proteins with survival to 90 in CHS. [file ACEL-23-e14136-s001.docx]

**Plasma Proteomic Signature of Human Longevity**

Supplementary Results

Table S1. List of proteins assayed on SOMAscan™ platform in CHS and AGES-Reykjavik

Table S2. Replication of 168 significant proteins associated with survival to 90 in CHS and AGES-Reykjavik

Table S3. Complete results of proteins (n = 4,985) associated with survival to 90 in CHS

Figure S1. Survival analysis of proteins associated with overall survival by Cox model. (A) Hazard ratios of top 30 significant proteins associated with overall survival in CHS. (B) Volcano plot summarizing the associations of all proteins (n=4,985) with overall survival in CHS. (C) Hazard ratios of top 30 significant proteins associated with overall survival in AGES-Reykjavik. (D) Volcano plot summarizing the associations of all proteins (n=4,783) with overall survival in AGES-Reykjavik.

Table S4. Difference of the significant proteins for survival to 90 and overall survival in CHS and AGES-Reykjavik.

Figure S2. Venn diagram showing the difference of the significant proteins for chronological age, survival to 90, and overall survival in CHS.

Figure S3. Sex-specific associations of top 50 proteins with survival to 90 in CHS

Table S5. Coefficients of the proteins chosen by LASSO

Table S6. Average causal mediation effect (ACME) of 4 functional measurements for partially mediating the associations of proteins with survival to 90.

Table S1. List of proteins assayed on SOMAscan™ platform in CHS and AGES-Reykjavik

Table S3. Complete results of proteins (n = 4,985) associated with survival to 90 in CHS

See online Excel Files

Table S2. Replication of 168 significant proteins associated with survival to 90 in CHS and AGES-Reykjavik

| SeqId | SomaId | UniProt | Target | Target full name | CHS | | AGES | |
| --- | --- | --- | --- | --- | --- | --- | --- | --- |
|  |  |  |  |  | OR (95% CI) | p-value | OR (95% CI) | p-value |
| 4374-45 | SL003869 | Q99988 | GDF-15 | growth differentiation factor 15 | 0.62 (0.56, 0.68) | 8.14E-24 | 0.6 (0.551, 0.65) | 1.01E-37 |
| 7655-11 | SL002785 | P16860 | N-terminal pro-BNP | natriuretic peptide B | 0.65 (0.60, 0.71) | 6.25E-22 | 0.66 (0.618, 0.71) | 5.67E-28 |
| 2677-1 | SL002644 | P00533 | ERBB1 | epidermal growth factor receptor | 1.49 (1.36, 1.62) | 7.67E-19 | 1.1 (1.026, 1.17) | 6.58E-03 |
| 3339-33 | SL007206 | P35442 | TSP2 | thrombospondin 2 | 0.69 (0.64, 0.75) | 9.22E-18 | 0.75 (0.698, 0.8) | 4.25E-17 |
| 4496-60 | SL000522 | P39900 | MMP-12 | matrix metallopeptidase 12 (macrophage elastase) | 0.69 (0.64, 0.76) | 2.93E-16 | 0.74 (0.692, 0.8) | 5.03E-16 |
| 7210-25 | SL004470 | P51693 | Amyloid-like protein 1 | amyloid beta (A4) precursor-like protein 1 | 1.41 (1.29, 1.54) | 7.30E-15 | 1.09 (1.016, 1.16) | 1.57E-02 |
| 8841-65 | SL008847 | Q8IUL8 | CILP2 | cartilage intermediate layer protein 2 | 1.38 (1.27, 1.50) | 1.51E-14 | 1.18 (1.107, 1.27) | 8.23E-07 |
| 2201-17 | SL000403 | P39060 | Endostatin | collagen, type XVIII, alpha 1 | 0.73 (0.67, 0.79) | 4.19E-14 | 0.72 (0.673, 0.78) | 7.00E-18 |
| 8252-2 | SL012580 | Q6P988 | NOTUM | notum pectinacetylesterase homolog (Drosophila) | 1.36 (1.25, 1.47) | 1.69E-13 | 1.15 (1.079, 1.23) | 2.87E-05 |
| 8969-49 | SL018823 | P08571 | CD14 | CD14 molecule | 0.73 (0.67, 0.79) | 2.92E-13 | 0.89 (0.826, 0.96) | 4.12E-03 |
| 2961-1 | SL000048 | P04070 | Protein C | protein C (inactivator of coagulation factors Va and VIIIa) | 1.35 (1.24, 1.46) | 7.12E-13 | 1.22 (1.131, 1.31) | 1.68E-07 |
| 12987-12 | SL019472 | Q16629 | SRSF7 | serine/arginine-rich splicing factor 7 | 0.72 (0.66, 0.79) | 1.02E-12 | 0.76 (0.705, 0.81) | 2.26E-14 |
| 9234-8 | SL009021 | Q9GZX9 | TWSG1 | twisted gastrulation homolog 1 (Drosophila) | 0.72 (0.66, 0.79) | 1.25E-12 | 0.86 (0.809, 0.92) | 1.45E-05 |
| 13463-1 | SL007573 | Q92626 | PXDN | peroxidasin homolog (Drosophila) | 0.73 (0.67, 0.80) | 1.66E-12 | 0.72 (0.665, 0.77) | 9.30E-19 |
| 3485-28 | SL000283 | P61769 | b2-Microglobulin | beta-2-microglobulin | 0.73 (0.67, 0.80) | 1.89E-12 | 0.69 (0.638, 0.74) | 1.87E-22 |
| 7211-2 | SL005355 | P07998 | Rnase 1 | ribonuclease, Rnase A family, 1 (pancreatic) | 0.73 (0.66, 0.79) | 2.25E-12 | 0.7 (0.65, 0.76) | 2.33E-20 |
| 5843-60 | SL003542 | Q96KQ7 | NG36 | euchromatic histone-lysine N-methyltransferase 2 | 1.35 (1.24, 1.47) | 2.50E-12 | 1.21 (1.125, 1.29) | 1.34E-07 |
| 5364-7 | SL007336 | Q01105 | SET | SET nuclear oncogene | 1.33 (1.23, 1.44) | 3.26E-12 | 0.96 (0.903, 1.03) | 2.93E-01 |
| 6259-60 | SL004822 | P61916 | Epididymal secretory protein E1 | Niemann-Pick disease, type C2 | 0.75 (0.69, 0.82) | 6.13E-12 | 0.8 (0.748, 0.86) | 2.11E-10 |
| 6036-78 | SL014294 | Q5JZY3 | EPHAA | EPH receptor A10 | 0.73 (0.66, 0.80) | 8.00E-12 | 0.74 (0.687, 0.79) | 1.30E-16 |
| 11388-75 | SL001690 | Q14508 | HE4 | WAP four-disulfide core domain 2 | 0.73 (0.67, 0.80) | 9.72E-12 | 0.65 (0.597, 0.7) | 3.82E-29 |
| 2602-2 | SL001996 | O15123 | Angiopoietin-2 | angiopoietin 2 | 0.76 (0.70, 0.82) | 1.43E-11 | 0.68 (0.637, 0.73) | 2.22E-25 |
| 6379-62 | SL012648 | Q86TH1 | ATL2 | ADAMTS-like 2 | 0.75 (0.69, 0.82) | 1.50E-11 | 0.79 (0.73, 0.85) | 3.36E-10 |
| 3152-57 | SL001800 | P20333 | TNF sR-II | tumor necrosis factor receptor superfamily, member 1B | 0.76 (0.70, 0.82) | 5.18E-11 | 0.79 (0.734, 0.84) | 2.64E-12 |
| 6294-11 | SL017389 | Q86Y30 | BAGE2 | B melanoma antigen family, member 2 | 0.75 (0.69, 0.82) | 5.47E-11 | 0.76 (0.711, 0.82) | 2.27E-14 |
| 12373-73 | SL014875 | P62995 | TRA2B | transformer 2 beta homolog (Drosophila) | 0.75 (0.69, 0.82) | 5.89E-11 | 0.75 (0.7, 0.81) | 9.30E-16 |
| 10521-10 | SL017989 | Q9BRK3 | MXRA8 | matrix-remodelling associated 8 | 1.33 (1.22, 1.45) | 5.96E-11 | 1.06 (0.987, 1.14) | 1.08E-01 |
| 2609-59 | SL001777 | P01034 | Cystatin C | cystatin C | 0.75 (0.68, 0.82) | 6.54E-11 | 0.69 (0.64, 0.75) | 4.25E-20 |
| 8480-29 | SL006527 | Q12805 | FBLN3 | EGF containing fibulin-like extracellular matrix protein 1 | 0.76 (0.70, 0.83) | 1.55E-10 | 0.79 (0.731, 0.85) | 7.82E-10 |
| 12988-49 | SL007342 | Q01844 | EWS | Ewing sarcoma breakpoint region 1 | 0.75 (0.69, 0.82) | 1.57E-10 | 0.74 (0.685, 0.79) | 1.07E-17 |
| 6544-33 | SL012542 | Q92832 | NELL1 | NEL-like 1 (chicken) | 1.29 (1.19, 1.40) | 2.37E-10 | 1.17 (1.092, 1.25) | 5.67E-06 |
| 6605-17 | SL005608 | P35858 | IGFALS | insulin-like growth factor binding protein, acid labile subunit | 1.31 (1.20, 1.43) | 3.45E-10 | 1.13 (1.06, 1.21) | 2.52E-04 |
| 10702-1 | SL012521 | Q2UY09 | COSA1 | collagen, type XXVIII, alpha 1 | 0.76 (0.70, 0.83) | 3.80E-10 | 0.68 (0.63, 0.73) | 3.99E-24 |
| 5852-6 | SL004783 | P80511 | S100A12 | S100 calcium binding protein A12 | 0.77 (0.71, 0.83) | 3.80E-10 | 0.9 (0.844, 0.96) | 1.71E-03 |
| 8304-50 | SL002539 | O00300 | OPG | tumor necrosis factor receptor superfamily, member 11b | 0.77 (0.71, 0.83) | 4.10E-10 | 0.72 (0.664, 0.77) | 9.14E-19 |
| 12661-44 | SL019788 | Q9H0R8 | GBRL1 | GABA(A) receptor-associated protein like 1 | 0.75 (0.69, 0.82) | 5.33E-10 | 0.72 (0.667, 0.77) | 2.35E-19 |
| 11214-40 | SL019363 | Q9UBS3 | DNJB9 | DnaJ (Hsp40) homolog, subfamily B, member 9 | 0.77 (0.71, 0.84) | 5.70E-10 | 0.83 (0.775, 0.89) | 4.96E-08 |
| 3322-52 | SL010464 | Q6UXM1 | LRIG3 | leucine-rich repeats and immunoglobulin-like domains 3 | 1.29 (1.19, 1.40) | 6.45E-10 | 1.05 (0.976, 1.12) | 2.07E-01 |
| 5701-81 | SL001871 | P05452 | Tetranectin | C-type lectin domain family 3, member B | 1.29 (1.19, 1.40) | 7.52E-10 | 1.37 (1.263, 1.49) | 4.31E-14 |
| 6388-21 | SL017451 | Q96EE4 | CC126 | coiled-coil domain containing 126 | 1.29 (1.19, 1.41) | 1.02E-09 | 1.17 (1.092, 1.25) | 8.42E-06 |
| 8885-6 | SL018710 | Q8IZS8 | CA2D3 | calcium channel, voltage-dependent, alpha 2/delta subunit 3 | 1.29 (1.19, 1.40) | 1.24E-09 | 1.21 (1.131, 1.3) | 5.02E-08 |
| 11196-31 | SL004928 | P12111 | Collagen alpha-3(VI) | collagen, type VI, alpha 3 | 0.76 (0.70, 0.83) | 1.41E-09 | 0.69 (0.645, 0.75) | 1.39E-22 |
| 2654-19 | SL001992 | P19438 | TNF sR-I | tumor necrosis factor receptor superfamily, member 1A | 0.77 (0.71, 0.84) | 2.27E-09 | 0.8 (0.743, 0.85) | 7.28E-11 |
| 4157-2 | SL000586 | P00734 | Thrombin | coagulation factor II (thrombin) | 1.28 (1.18, 1.39) | 2.68E-09 | 1.04 (0.978, 1.11) | 1.98E-01 |
| 8368-102 | SL001800 | P20333 | TNF sR-II | tumor necrosis factor receptor superfamily, member 1B | 0.78 (0.72, 0.85) | 3.04E-09 | 0.73 (0.677, 0.78) | 8.52E-18 |
| 14037-18 | SL013273 | Q9H6Z4 | RANB3 | RAN binding protein 3 | 0.78 (0.72, 0.85) | 3.15E-09 | 0.75 (0.702, 0.81) | 1.33E-14 |
| 11178-21 | SL012540 | Q4LDE5 | SVEP1 | sushi, von Willebrand factor type A, EGF and pentraxin domain containing 1 | 0.78 (0.72, 0.85) | 4.65E-09 | 0.67 (0.622, 0.72) | 1.77E-26 |
| 9021-1 | SL010386 | Q96D42 | TIM-1 | hepatitis A virus cellular receptor 1 | 0.78 (0.71, 0.85) | 5.16E-09 | 0.77 (0.722, 0.83) | 5.53E-14 |
| 9468-8 | SL005403 | Q12907 | Lectin, mannose-binding 2 | lectin, mannose-binding 2 | 0.78 (0.72, 0.85) | 7.54E-09 | 0.74 (0.69, 0.8) | 1.07E-15 |
| 12630-8 | SL019787 | P53365 | ARFP2 | ADP-ribosylation factor interacting protein 2 | 1.27 (1.17, 1.38) | 1.01E-08 | 1.08 (1.01, 1.16) | 2.41E-02 |
| 9316-67 | SL012843 | Q9HC57 | WFDC1 | WAP four-disulfide core domain 1 | 0.79 (0.73, 0.85) | 1.05E-08 | 0.79 (0.738, 0.85) | 3.97E-11 |
| 10903-50 | SL017987 | Q9UNK0 | STX8 | syntaxin 8 | 0.79 (0.73, 0.86) | 1.13E-08 | 0.71 (0.664, 0.77) | 3.40E-20 |
| 5717-2 | SL004657 | O75339 | CILP | cartilage intermediate layer protein, nucleotide pyrophosphohydrolase | 0.79 (0.73, 0.86) | 1.28E-08 | 0.74 (0.69, 0.79) | 6.05E-17 |
| 5029-3 | SL006528 | Q12884 | SEPR | fibroblast activation protein, alpha | 1.27 (1.17, 1.38) | 1.79E-08 | 1.08 (1.01, 1.15) | 2.36E-02 |
| 4874-3 | SL000003 | P03950 | Angiogenin | angiogenin, ribonuclease, Rnase A family, 5 | 0.79 (0.73, 0.86) | 2.06E-08 | 0.82 (0.763, 0.87) | 5.27E-09 |
| 8265-225 | SL013037 | P42167 | LAP2B | thymopoietin | 0.79 (0.72, 0.86) | 2.12E-08 | 0.71 (0.665, 0.77) | 2.34E-20 |
| 4920-10 | SL000510 | P61626 | Lysozyme | lysozyme | 0.79 (0.72, 0.86) | 2.93E-08 | 0.75 (0.694, 0.8) | 5.70E-15 |
| 5715-4 | SL017100 | Q9BUJ0 | ABHEA | abhydrolase domain containing 14A | 0.80 (0.74, 0.86) | 3.15E-08 | 0.76 (0.711, 0.82) | 2.26E-14 |
| 3079-62 | SL005152 | Q99969 | TIG2 | retinoic acid receptor responder (tazarotene induced) 2 | 0.79 (0.73, 0.86) | 3.60E-08 | 0.81 (0.747, 0.87) | 2.44E-08 |
| 6077-63 | SL013510 | Q9NZK5 | CECR1 | cat eye syndrome chromosome region, candidate 1 | 0.80 (0.74, 0.87) | 4.32E-08 | 0.84 (0.784, 0.9) | 1.76E-07 |
| 5646-20 | SL012758 | Q93091 | RNAS6 | ribonuclease, Rnase A family, k6 | 0.79 (0.73, 0.86) | 5.17E-08 | 0.74 (0.691, 0.79) | 5.24E-17 |
| 9380-2 | SL003846 | Q9BX93 | sPLA(2)-XIII | phospholipase A2, group XIIB | 1.24 (1.15, 1.35) | 5.67E-08 | 1.13 (1.053, 1.2) | 4.70E-04 |
| 3438-10 | SL009324 | O95633 | FSTL3 | follistatin-like 3 (secreted glycoprotein) | 0.79 (0.72, 0.86) | 5.80E-08 | 0.72 (0.671, 0.78) | 5.54E-18 |
| 5644-60 | SL007198 | P34096 | RNAS4 | ribonuclease, Rnase A family, 4 | 0.80 (0.74, 0.87) | 6.26E-08 | 0.74 (0.691, 0.8) | 1.04E-16 |
| 10464-6 | SL008696 | Q9H6X2 | ANTR1 | anthrax toxin receptor 1 | 1.26 (1.16, 1.36) | 6.65E-08 | 1.22 (1.136, 1.31) | 3.85E-08 |
| 2974-61 | SL004855 | Q12860 | contactin-1 | contactin 1 | 1.25 (1.15, 1.36) | 8.65E-08 | 0.97 (0.903, 1.03) | 3.27E-01 |
| 14151-4 | SL015510 | P05161 | UCRP | ISG15 ubiquitin-like modifier | 0.80 (0.74, 0.87) | 8.69E-08 | 0.81 (0.753, 0.86) | 3.20E-10 |
| 2475-1 | SL004010 | P10721 | SCF sR | v-kit Hardy-Zuckerman 4 feline sarcoma viral oncogene homolog | 1.25 (1.15, 1.35) | 8.79E-08 | 1.04 (0.974, 1.11) | 2.39E-01 |
| 9348-1 | SL010648 | Q9NZP8 | C1RL1 | complement component 1, r subcomponent-like | 0.81 (0.75, 0.87) | 9.92E-08 | 0.89 (0.82, 0.96) | 2.64E-03 |
| 5452-71 | SL008835 | P07306 | ASGR1 | asialoglycoprotein receptor 1 | 0.80 (0.74, 0.87) | 1.26E-07 | 1.02 (0.947, 1.09) | 6.71E-01 |
| 7953-20 | SL018318 | Q13291 | SLAF1 | signaling lymphocytic activation molecule family member 1 | 0.79 (0.73, 0.86) | 1.46E-07 | 0.82 (0.762, 0.87) | 7.70E-09 |
| 10565-19 | SL017951 | O94933 | SLIK3 | SLIT and NTRK-like family, member 3 | 1.25 (1.15, 1.36) | 1.64E-07 | 1.05 (0.981, 1.12) | 1.66E-01 |
| 10803-22 | SL018436 | Q9Y5G2 | PCDGE | protocadherin gamma subfamily B, 2 | 0.81 (0.74, 0.87) | 1.85E-07 | 0.81 (0.75, 0.87) | 3.92E-09 |
| 11212-7 | SL008805 | Q8NBS9 | TXND5 | thioredoxin domain containing 5 (endoplasmic reticulum) | 0.80 (0.74, 0.87) | 1.99E-07 | 0.74 (0.686, 0.79) | 5.01E-16 |
| 14337-1 | SL019699 | O43617 | TPPC3 | trafficking protein particle complex 3 | 0.79 (0.72, 0.86) | 2.08E-07 | 0.92 (0.859, 0.98) | 1.05E-02 |
| 10612-18 | SL008694 | O60568 | PLOD3 | procollagen-lysine, 2-oxoglutarate 5-dioxygenase 3 | 0.81 (0.75, 0.88) | 2.17E-07 | 0.87 (0.812, 0.93) | 5.51E-05 |
| 4834-61 | SL002654 | P29317 | Epithelial cell kinase | EPH receptor A2 | 0.81 (0.74, 0.87) | 2.76E-07 | 0.74 (0.685, 0.79) | 4.34E-16 |
| 6324-11 | SL012757 | Q92874 | DNSL2 | deoxyribonuclease I-like 2 | 0.80 (0.74, 0.87) | 2.78E-07 | 0.78 (0.725, 0.84) | 2.77E-11 |
| 6284-7 | SL012605 | Q6UX46 | F150B | family with sequence similarity 150, member B | 0.81 (0.75, 0.88) | 2.92E-07 | 0.75 (0.698, 0.8) | 1.04E-15 |
| 4153-11 | SL000091 | P07288, P01011 | alpha-1-antichymotrypsin complex | kallikrein-related peptidase 3 | 0.81 (0.75, 0.88) | 3.05E-07 | 0.9 (0.843, 0.96) | 1.69E-03 |
| 7251-64 | SL012818 | Q9BXJ4 | C1QT3 | C1q and tumor necrosis factor related protein 3 | 1.22 (1.13, 1.32) | 3.51E-07 | 1.05 (0.981, 1.12) | 1.58E-01 |
| 4297-62 | SL005115 | Q9HCB6 | Spondin-1 | spondin 1, extracellular matrix protein | 0.81 (0.75, 0.88) | 4.36E-07 | 0.73 (0.679, 0.78) | 1.91E-17 |
| 10445-20 | SL004747 | O95445 | ApoM | apolipoprotein M | 1.23 (1.13, 1.33) | 4.57E-07 | 1.17 (1.091, 1.26) | 1.47E-05 |
| 13950-9 | SL015375 | Q9Y5P4 | C43BP | collagen, type IV, alpha 3 (Goodpasture antigen) binding protein | 0.82 (0.75, 0.88) | 4.69E-07 | 0.67 (0.624, 0.72) | 3.92E-25 |
| 2948-58 | SL005168 | P10912 | Growth hormone receptor | growth hormone receptor | 1.26 (1.15, 1.38) | 5.37E-07 | 1.24 (1.148, 1.34) | 3.89E-08 |
| 9216-100 | SL009948 | O15031 | PLXB2 | plexin B2 | 0.82 (0.76, 0.89) | 6.42E-07 | 0.81 (0.752, 0.86) | 7.53E-10 |
| 4498-62 | SL003764 | P13591 | NCAM-120 | neural cell adhesion molecule 1 | 1.24 (1.14, 1.34) | 6.51E-07 | 0.94 (0.877, 1.01) | 9.96E-02 |
| 6291-55 | SL011368 | Q9BQT9 | Alcadein-beta | calsyntenin 3 | 0.81 (0.74, 0.88) | 7.13E-07 | 0.73 (0.675, 0.78) | 1.21E-17 |
| 5134-52 | SL007547 | Q8TDQ0 | TIMD3 | hepatitis A virus cellular receptor 2 | 0.82 (0.76, 0.89) | 8.09E-07 | 0.76 (0.709, 0.82) | 4.78E-14 |
| 2900-53 | SL003329 | Q16627 | HCC-1 | chemokine (C-C motif) ligand 14 | 0.82 (0.76, 0.89) | 8.71E-07 | 0.76 (0.713, 0.82) | 2.40E-14 |
| 6563-78 | SL017509 | Q9Y5H2 | PCDGB | protocadherin gamma subfamily A, 11 | 0.82 (0.75, 0.88) | 9.00E-07 | 0.77 (0.716, 0.83) | 1.57E-12 |
| 9296-15 | SL008499 | P23468 | PTPRD | protein tyrosine phosphatase, receptor type, D | 1.24 (1.14, 1.35) | 9.24E-07 | 1.02 (0.947, 1.09) | 6.65E-01 |
| 14227-21 | SL008040 | P14649 | MYL6B | myosin, light chain 6B, alkali, smooth muscle and non-muscle | 0.81 (0.75, 0.88) | 9.46E-07 | 0.8 (0.744, 0.86) | 3.92E-10 |
| 12727-7 | SL019877 | Q9P2B2 | FPRP | prostaglandin F2 receptor negative regulator | 1.22 (1.13, 1.33) | 9.90E-07 | 1.16 (1.085, 1.24) | 1.43E-05 |
| 6570-1 | SL017501 | Q5TAT6 | CODA1 | collagen, type XIII, alpha 1 | 1.24 (1.14, 1.36) | 1.02E-06 | 1.1 (1.021, 1.18) | 1.21E-02 |
| 10490-3 | SL011102 | P04843 | RPN1 | ribophorin I | 0.81 (0.74, 0.88) | 1.07E-06 | 0.85 (0.793, 0.91) | 3.93E-06 |
| 11573-3 | SL017909 | Q13247 | SRSF6 | serine/arginine-rich splicing factor 6 | 0.80 (0.73, 0.88) | 1.26E-06 | 0.84 (0.783, 0.9) | 6.15E-07 |
| 11109-56 | SL012540 | Q4LDE5 | SVEP1 | sushi, von Willebrand factor type A, EGF and pentraxin domain containing 1 | 0.81 (0.75, 0.89) | 1.28E-06 | 0.67 (0.62, 0.72) | 1.39E-26 |
| 10666-7 | SL012791 | Q9UJJ9 | GNPTG | N-acetylglucosamine-1-phosphate transferase, gamma subunit | 0.82 (0.76, 0.89) | 1.35E-06 | 0.94 (0.871, 1.01) | 1.14E-01 |
| 2944-66 | SL005156 | P41271 | DAN | neuroblastoma, suppression of tumorigenicity 1 | 0.80 (0.73, 0.88) | 1.41E-06 | 0.74 (0.683, 0.79) | 2.10E-16 |
| 9266-1 | SL004968 | Q9NP99 | sTREM-1 | triggering receptor expressed on myeloid cells 1 | 0.81 (0.75, 0.88) | 1.42E-06 | 0.74 (0.693, 0.8) | 9.04E-16 |
| 2658-27 | SL004639 | Q16288 | TrkC | neurotrophic tyrosine kinase, receptor, type 3 | 1.22 (1.13, 1.33) | 1.43E-06 | 0.99 (0.918, 1.06) | 6.85E-01 |
| 9231-23 | SL017105 | Q9NX62 | IMPA3 | inositol monophosphatase domain containing 1 | 1.23 (1.13, 1.33) | 1.44E-06 | 1.02 (0.953, 1.09) | 5.87E-01 |
| 10833-64 | SL012788 | Q96QV1 | HHIP | hedgehog interacting protein | 1.22 (1.13, 1.32) | 1.50E-06 | 1.06 (0.993, 1.14) | 7.76E-02 |
| 3029-52 | SL005157 | Q9NNX6 | DC-SIGN | CD209 molecule | 1.21 (1.12, 1.31) | 1.52E-06 | 1.09 (1.019, 1.16) | 1.18E-02 |
| 6927-7 | SL017913 | P52848 | NDST1 | N-deacetylase/N-sulfotransferase (heparan glucosaminyl) 1 | 1.22 (1.12, 1.32) | 1.57E-06 | 1.01 (0.944, 1.08) | 7.78E-01 |
| 8957-72 | SL012771 | Q96DZ1 | XTP3B | endoplasmic reticulum lectin 1 | 0.78 (0.70, 0.86) | 1.57E-06 | 0.73 (0.677, 0.78) | 9.39E-18 |
| 3651-50 | SL003201 | P35968 | VEGF sR2 | kinase insert domain receptor (a type III receptor tyrosine kinase) | 1.21 (1.12, 1.31) | 1.66E-06 | 1.1 (1.026, 1.17) | 6.97E-03 |
| 11361-73 | SL000155 | P19971 | TP | thymidine phosphorylase | 0.83 (0.76, 0.89) | 1.72E-06 | 0.97 (0.904, 1.03) | 3.32E-01 |
| 13943-38 | SL020276 | Q9C005 | DPY30 | dpy-30 homolog (C. elegans) | 0.82 (0.76, 0.89) | 1.85E-06 | 0.76 (0.707, 0.81) | 1.97E-14 |
| 9278-9 | SL009893 | Q99645 | Epiphycan | epiphycan | 0.83 (0.76, 0.89) | 1.91E-06 | 0.76 (0.702, 0.81) | 1.44E-13 |
| 3038-9 | SL003326 | O14625 | I-TAC | chemokine (C-X-C motif) ligand 11 | 0.83 (0.76, 0.89) | 2.09E-06 | 0.85 (0.795, 0.91) | 2.26E-06 |
| 9326-33 | SL004711 | P19823 | ITI heavy chain H2 | inter-alpha (globulin) inhibitor H2 | 1.21 (1.12, 1.31) | 2.41E-06 | 1.07 (1.004, 1.15) | 3.88E-02 |
| 5339-49 | SL004477 | P06702 | calgranulin B | S100 calcium binding protein A9 | 0.83 (0.77, 0.90) | 2.57E-06 | 0.9 (0.838, 0.96) | 1.25E-03 |
| 12549-33 | SL014636 | O60760 | PTGD2 | hematopoietic prostaglandin D synthase | 1.21 (1.12, 1.30) | 2.72E-06 | 1.14 (1.066, 1.22) | 1.15E-04 |
| 4148-49 | SL002755 | Q13219 | PAPP-A | pregnancy-associated plasma protein A, pappalysin 1 | 0.81 (0.74, 0.89) | 3.03E-06 | 0.78 (0.725, 0.83) | 3.47E-12 |
| 13242-134 | SL019995 | Q9BYB0 | SHAN3 | SH3 and multiple ankyrin repeat domains 3 | 0.83 (0.76, 0.90) | 3.27E-06 | 0.84 (0.79, 0.9) | 8.75E-07 |
| 7861-9 | SL008955 | Q01974 | ROR2 | receptor tyrosine kinase-like orphan receptor 2 | 0.81 (0.74, 0.88) | 3.45E-06 | 0.77 (0.719, 0.82) | 9.07E-14 |
| 5738-25 | SL007889 | P11686 | SP-C | surfactant protein C | 0.83 (0.77, 0.90) | 4.02E-06 | 0.78 (0.725, 0.83) | 3.16E-13 |
| 5688-65 | SL012852 | Q9NTU7 | CBLN4 | cerebellin 4 precursor | 1.20 (1.11, 1.30) | 4.04E-06 | 1.03 (0.964, 1.1) | 3.75E-01 |
| 6556-5 | SL012863 | Q9UJA9 | ENPP5 | ectonucleotide pyrophosphatase/phosphodiesterase 5 (putative) | 1.22 (1.12, 1.32) | 4.70E-06 | 1.05 (0.98, 1.13) | 1.57E-01 |
| 3449-58 | SL004876 | P29622 | Kallistatin | serpin peptidase inhibitor, clade A (alpha-1 antiproteinase, antitrypsin), member 4 | 1.20 (1.11, 1.30) | 4.77E-06 | 1.11 (1.034, 1.18) | 3.62E-03 |
| 9525-1 | SL013388 | Q13308 | PTK7 | PTK7 protein tyrosine kinase 7 | 0.84 (0.77, 0.90) | 4.92E-06 | 0.8 (0.748, 0.86) | 2.55E-10 |
| 9341-1 | SL014328 | Q9GZP0 | PDGFD | platelet derived growth factor D | 0.83 (0.77, 0.90) | 5.03E-06 | 0.98 (0.918, 1.05) | 5.85E-01 |
| 5129-12 | SL005221 | Q14162 | SREC-I | scavenger receptor class F, member 1 | 0.83 (0.77, 0.90) | 5.37E-06 | 0.81 (0.761, 0.87) | 3.36E-09 |
| 2692-74 | SL002528 | P14555 | NPS-PLA2 | phospholipase A2, group IIA (platelets, synovial fluid) | 0.83 (0.76, 0.90) | 5.58E-06 | 0.86 (0.8, 0.91) | 4.48E-06 |
| 11516-7 | SL006131 | P07148 | FABPL | fatty acid binding protein 1, liver | 0.83 (0.76, 0.90) | 5.76E-06 | 0.82 (0.771, 0.88) | 1.54E-08 |
| 8953-47 | SL013046 | P51858 | HDGF | hepatoma-derived growth factor | 0.83 (0.77, 0.90) | 5.91E-06 | 0.75 (0.704, 0.81) | 1.05E-15 |
| 2617-56 | SL002519 | P21860 | ERBB3 | v-erb-b2 erythroblastic leukemia viral oncogene homolog 3 (avian) | 1.22 (1.12, 1.33) | 5.93E-06 | 0.99 (0.924, 1.06) | 7.85E-01 |
| 4246-40 | SL004154 | P32004 | NCAM-L1 | L1 cell adhesion molecule | 1.20 (1.11, 1.29) | 6.20E-06 | 1.02 (0.958, 1.1) | 4.72E-01 |
| 9580-5 | SL005798 | Q13753 | Laminin gamma-2 | laminin, gamma 2 | 0.83 (0.76, 0.90) | 6.32E-06 | 0.78 (0.727, 0.83) | 1.54E-12 |
| 4811-33 | SL004739 | Q14624 | ITI heavy chain H4 | inter-alpha (globulin) inhibitor H4 (plasma Kallikrein-sensitive glycoprotein) | 0.83 (0.77, 0.90) | 6.70E-06 | 0.87 (0.811, 0.94) | 1.82E-04 |
| 7957-2 | SL012750 | Q8WXD2 | SCG3 | secretogranin III | 1.21 (1.11, 1.32) | 7.18E-06 | 0.93 (0.866, 0.99) | 2.82E-02 |
| 4968-50 | SL008099 | P40121 | CAPG | capping protein (actin filament), gelsolin-like | 0.83 (0.76, 0.90) | 7.22E-06 | 0.83 (0.774, 0.89) | 9.99E-08 |
| 3060-43 | SL000325 | P02748 | C9 | complement component 9 | 0.81 (0.74, 0.89) | 7.91E-06 | 0.79 (0.737, 0.85) | 2.42E-11 |
| 5315-22 | SL000052 | P45379 | Troponin T | troponin T type 2 (cardiac) | 0.82 (0.75, 0.89) | 7.98E-06 | 1.14 (1.063, 1.22) | 2.27E-04 |
| 4467-49 | SL005488 | Q14515 | SPARCL1 | SPARC-like 1 (hevin) | 1.20 (1.11, 1.29) | 8.34E-06 | 0.96 (0.901, 1.03) | 2.73E-01 |
| 13565-2 | SL003760 | Q08999 | p130 | retinoblastoma-like 2 (p130) | 0.84 (0.77, 0.90) | 8.48E-06 | 0.8 (0.746, 0.86) | 7.90E-10 |
| 8469-41 | SL000466 | P18065 | IGFBP-2 | insulin-like growth factor binding protein 2, 36kDa | 0.81 (0.73, 0.89) | 9.12E-06 | 0.68 (0.627, 0.74) | 6.20E-19 |
| 10721-76 | SL018779 | Q9UN71 | PCDGG | protocadherin gamma subfamily B, 4 | 0.84 (0.77, 0.91) | 1.07E-05 | 0.94 (0.882, 1.01) | 9.24E-02 |
| 2643-57 | SL004183 | P22223 | P-Cadherin | cadherin 3, type 1, P-cadherin (placental) | 1.19 (1.10, 1.29) | 1.10E-05 | 1.12 (1.045, 1.2) | 1.35E-03 |
| 3024-18 | SL000250 | P08697 | a2-Antiplasmin | serpin peptidase inhibitor, clade F (alpha-2 antiplasmin, pigment epithelium derived factor), member 2 | 1.20 (1.11, 1.31) | 1.11E-05 | 1.3 (1.198, 1.41) | 3.15E-10 |
| 6260-14 | SL017414 | Q96GL9 | F163A | family with sequence similarity 163, member A | 0.84 (0.78, 0.91) | 1.12E-05 | 0.78 (0.728, 0.84) | 1.43E-12 |
| 5353-89 | SL001990 | P18510 | IL-1Ra | interleukin 1 receptor antagonist | 0.83 (0.76, 0.90) | 1.12E-05 | 1.04 (0.968, 1.11) | 3.09E-01 |
| 8484-24 | SL000498 | P41159 | Leptin | leptin | 1.34 (1.18, 1.53) | 1.12E-05 | 1.07 (0.958, 1.2) | 2.29E-01 |
| 5903-91 | SL004899 | P11142 | HSP70 protein 8 | heat shock 70kDa protein 8 | 0.83 (0.77, 0.90) | 1.21E-05 | 0.86 (0.806, 0.92) | 1.83E-05 |
| 4152-58 | SL000545 | P03952 | Prekallikrein | kallikrein B, plasma (Fletcher factor) 1 | 1.20 (1.11, 1.31) | 1.22E-05 | 1.14 (1.066, 1.23) | 1.85E-04 |
| 4413-3 | SL001888 | P03973 | SLPI | secretory leukocyte peptidase inhibitor | 0.83 (0.77, 0.90) | 1.23E-05 | 0.72 (0.664, 0.77) | 1.82E-18 |
| 5632-6 | SL012846 | Q9NQ79 | CRAC1 | cartilage acidic protein 1 | 1.19 (1.10, 1.29) | 1.24E-05 | 1.09 (1.019, 1.17) | 1.29E-02 |
| 3348-49 | SL003994 | P13497 | BMP-1 | bone morphogenetic protein 1 | 1.19 (1.10, 1.29) | 1.33E-05 | 1.18 (1.096, 1.26) | 7.00E-06 |
| 10749-18 | SL012818 | Q9BXJ4 | C1QT3 | C1q and tumor necrosis factor related protein 3 | 0.83 (0.77, 0.90) | 1.39E-05 | 0.83 (0.769, 0.89) | 1.86E-07 |
| 4880-21 | SL007756 | Q9UK05 | GDF2 | growth differentiation factor 2 | 1.20 (1.11, 1.31) | 1.44E-05 | 0.99 (0.924, 1.05) | 6.62E-01 |
| 11347-9 | SL006113 | P37837 | Transaldolase | transaldolase 1 | 0.84 (0.78, 0.91) | 1.46E-05 | 0.91 (0.853, 0.98) | 7.59E-03 |
| 3378-49 | SL000064 | P49862 | Kallikrein 7 | kallikrein-related peptidase 7 | 1.19 (1.10, 1.29) | 1.47E-05 | 1.03 (0.967, 1.11) | 3.28E-01 |
| 5628-21 | SL012850 | Q9NS98 | SEM3G | sema domain, immunoglobulin domain (Ig), short basic domain, secreted, (emaphoring) 3G | 1.19 (1.10, 1.29) | 1.54E-05 | 0.99 (0.927, 1.06) | 7.72E-01 |
| 6296-36 | SL017395 | Q8WWZ8 | OIT3 | oncoprotein induced transcript 3 | 0.83 (0.76, 0.90) | 1.54E-05 | 0.76 (0.705, 0.81) | 7.91E-15 |
| 13392-13 | SL008814 | P05026 | AT1B1 | ATPase, Na+/K+ transporting, beta 1 polypeptide | 1.20 (1.11, 1.31) | 1.55E-05 | 1.23 (1.144, 1.32) | 1.59E-08 |
| 5456-59 | SL006694 | Q96KN2 | CNDP1 | carnosine dipeptidase 1 (metallopeptidase M20 family) | 1.19 (1.10, 1.30) | 1.59E-05 | 1.1 (1.03, 1.18) | 4.62E-03 |
| 12831-21 | SL015132 | Q96BS2 | TESC | tescalcin | 0.84 (0.78, 0.91) | 1.60E-05 | 0.91 (0.855, 0.98) | 6.90E-03 |
| 6276-16 | SL005252 | P09958 | Furin | furin (paired basic amino acid cleaving enzyme) | 0.83 (0.76, 0.90) | 1.61E-05 | 1.19 (1.11, 1.27) | 8.76E-07 |
| 5000-52 | SL006522 | Q08380 | LG3BP | lectin, galactoside-binding, soluble, 3 binding protein | 0.84 (0.78, 0.91) | 1.68E-05 | 0.87 (0.813, 0.93) | 5.28E-05 |
| 10832-24 | SL018597 | Q9UBX8 | B4GT6 | UDP-Gal:betaGlcNAc beta 1,4- galactosyltransferase, polypeptide 6 | 1.20 (1.11, 1.31) | 1.70E-05 | 1.16 (1.085, 1.24) | 1.52E-05 |
| 2789-26 | SL000525 | P09237 | MMP-7 | matrix metallopeptidase 7 (matrilysin, uterine) | 0.84 (0.78, 0.91) | 1.75E-05 | 0.68 (0.631, 0.73) | 1.06E-27 |
| 8039-41 | SL018368 | Q8N128 | F177A | family with sequence similarity 177, member A1 | 1.20 (1.10, 1.30) | 1.78E-05 | 1.04 (0.975, 1.11) | 2.24E-01 |
| 5542-22 | SL006397 | O14786 | NRP1 | neuropilin 1 | 0.84 (0.77, 0.91) | 1.80E-05 | 0.84 (0.783, 0.91) | 8.00E-06 |
| 13624-17 | SL020213 | O95544 | NADK | NAD kinase | 0.83 (0.76, 0.90) | 1.95E-05 | 0.92 (0.863, 0.99) | 2.12E-02 |
| 10418-36 | SL011111 | Q86Y82 | STX12 | syntaxin 12 | 0.84 (0.77, 0.91) | 1.97E-05 | 0.9 (0.844, 0.97) | 3.00E-03 |
| 8300-82 | SL018446 | O75381 | PEX14 | peroxisomal biogenesis factor 14 | 0.84 (0.77, 0.91) | 1.98E-05 | 0.98 (0.92, 1.05) | 6.21E-01 |

Note: Estimates were from logistic regression adjusted for age, sex, race, height, weight, waist, smoking status, and pack years. Significant threshold was 2.0×10^-5^.

Figure S1. Survival analysis of proteins associated with overall survival by Cox model. (A) Hazard ratios of top 30 significant proteins associated with overall survival in CHS. (B) Volcano plot summarizing the associations of all proteins (n=4,985) with overall survival in CHS. (C) Hazard ratios of top 30 significant proteins associated with overall survival in AGES-Reykjavik. (D) Volcano plot summarizing the associations of all proteins (n=4,783) with overall survival in AGES-Reykjavik. Beta coefficients and p-values are from Cox regression adjusted for age, sex, race, height, weight, waist, smoking status and pack years. Significant proteins (p-value< 2.0×10^-5^) are shown as red dots and top proteins are annotated.


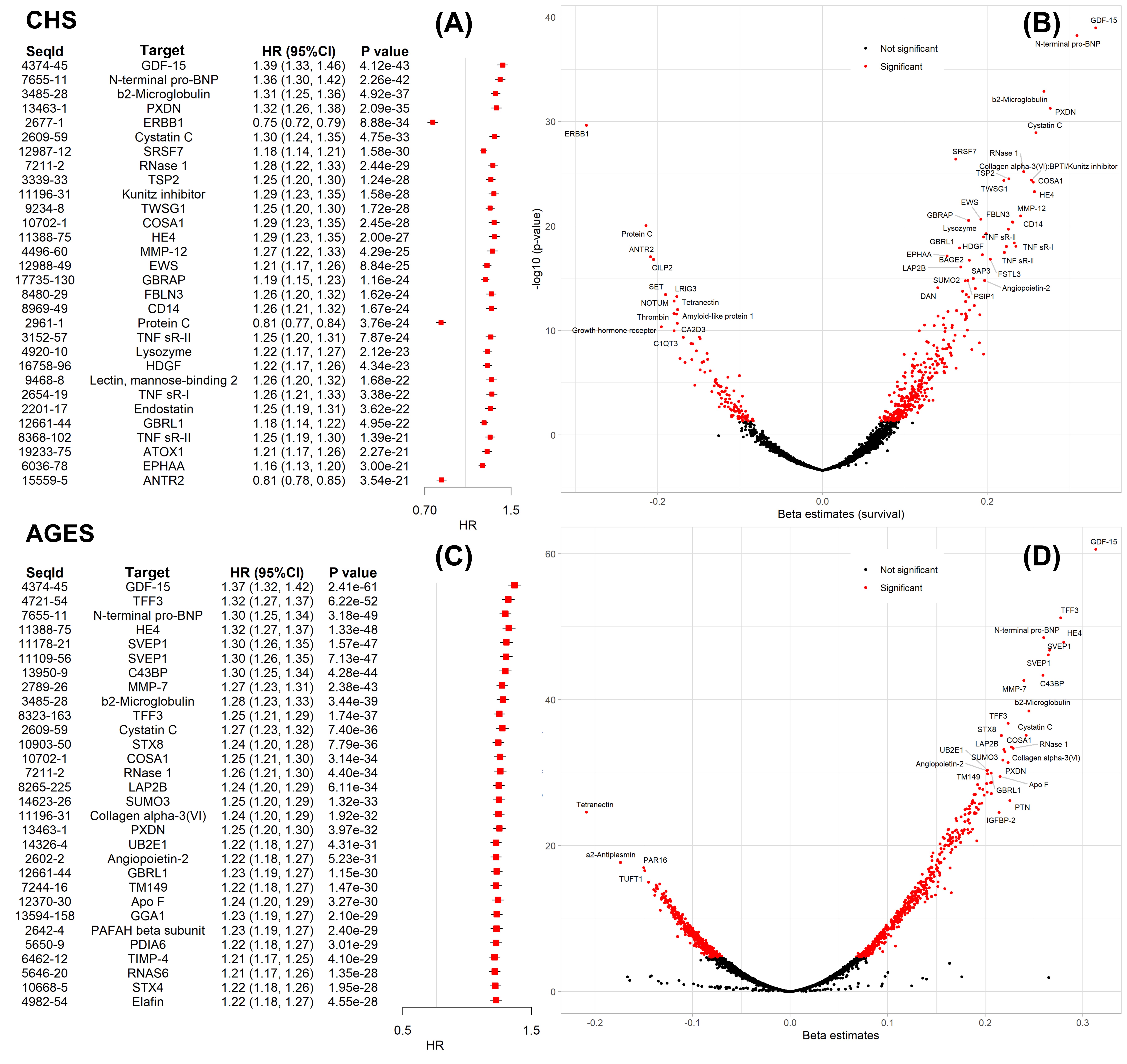


Table S4. Difference of the significant proteins for survival to 90 versus overall survival in CHS and AGES

| SeqId | SomaId | UniProt | Target | Target full name | Survival to 90 | | Overall survival | |
| --- | --- | --- | --- | --- | --- | --- | --- | --- |
|  |  |  |  |  | OR (95% CI) | p-value | HR (95% CI) | p-value |
| CHS |  |  |  |  |  |  |  |  |
| 3060-43 | SL000325 | P02748 | C9 | Complement component C9 | 0.81 (0.74, 0.89) | 7.91E-06 | 1.12 (1.06, 1.17) | 2.81E-05 |
| 16060-99 | SL008193 | Q14112 | NID2 | Nidogen-2 | 0.85 (0.78, 0.91) | 1.83E-05 | 1.08 (1.04, 1.13) | 5.73E-05 |
| 5000-52 | SL006522 | Q08380 | LG3BP | Galectin-3-binding protein | 0.84 (0.78, 0.91) | 1.68E-05 | 1.09 (1.04, 1.14) | 5.81E-05 |
| 7957-2 | SL012750 | Q8WXD2 | SCG3 | Secretogranin-3 | 1.21 (1.11, 1.32) | 7.18E-06 | 0.92 (0.88, 0.96) | 0.000203 |
| 4880-21 | SL007756 | Q9UK05 | GDF2 | Growth/differentiation factor 2 | 1.20 (1.11, 1.31) | 1.44E-05 | 0.92 (0.88, 0.96) | 0.000229 |
| 15556-49 | SL005393 | P19961 | Alpha-amylase 2B | Alpha-amylase 2B | 1.20 (1.10, 1.30) | 1.86E-05 | 0.92 (0.88, 0.96) | 0.000249 |
| 8484-24 | SL000498 | P41159 | Leptin | Leptin | 1.34 (1.18, 1.53) | 1.12E-05 | 0.88 (0.82, 0.95) | 0.000483 |
| 19563-3 | SL008781 | Q9BYH1 | SEZ6L | Seizure 6-like protein | 1.22 (1.12, 1.33) | 5.55E-06 | 0.92 (0.88, 0.96) | 0.000493 |
| 15511-37 | SL008970 | O95502 | NPTXR | Neuronal pentraxin receptor | 1.26 (1.16, 1.36) | 5.31E-08 | 0.92 (0.88, 0.97) | 0.000556 |
| AGES |  |  |  |  |  |  |  |  |
| 8654-13 | SL004641 | O14672 | ADAM 10 | ADAM metallopeptidase domain 10 | 1.17 (1.09, 1.25) | 6.92e-06 | 0.93 (0.91, 0.97) | 6.98e-05 |
| 4961-17 | SL004209 | P07355 | annexin II | annexin A2 | 0.85 (0.80, 0.91) | 7.30e-06 | 1.07 (1.04, 1.11) | 5.23e-05 |
| 6622-90 | SL011868 | Q9ULZ1 | APEL | apelin | 1.18 (1.09, 1.27) | 1.67e-05 | 0.92 (0.89, 0.96) | 3.65e-05 |
| 7105-7 | SL018130 | Q9BTV7 | CABL2 | Cdk5 and Abl enzyme substrate 2 | 1.17 (1.09, 1.25) | 7.27e-06 | 0.94 (0.91, 0.97) | 3.54e-04 |
| 3299-29 | SL010455 | O94779 | Contactin-5 | contactin 5 | 1.18 (1.09, 1.27) | 1.88e-05 | 0.92 (0.89, 0.96) | 2.08e-05 |
| 12904-180 | SL004440 | O60494 | cubilin | cubilin (intrinsic factor-cobalamin receptor) | 1.19 (1.11, 1.28) | 2.36e-06 | 0.93 (0.89, 0.96) | 2.36e-05 |
| 5679-16 | SL004534 | P81534 | HBD-3 | defensin, beta 103B | 1.17 (1.1, 1.26) | 7.31e-06 | 0.94 (0.91, 0.97) | 1.26e-04 |
| 3607-71 | SL009412 | Q9UBP4 | DKK3 | dickkopf homolog 3 (Xenopus laevis) | 0.85 (0.79, 0.91) | 1.11e-05 | 1.07 (1.03, 1.11) | 1.22e-04 |
| 9536-16 | SL012501 | Q14507 | EP3A | epididymal protein 3A | 1.22 (1.12, 1.32) | 3.04e-06 | 0.92 (0.88, 0.96) | 4.59e-05 |
| 4706-17 | SL010830 | P11171 | 41 | erythrocyte membrane protein band 4.1 (elliptocytosis 1, RH-linked) | 1.16 (1.09, 1.24) | 9.15e-06 | 0.94 (0.91, 0.97) | 8.60e-05 |
| 3805-16 | SL010458 | Q9NQ30 | Endocan | endothelial cell-specific molecule 1 | 0.86 (0.80, 0.92) | 1.15e-05 | 1.07 (1.04, 1.11) | 5.19e-05 |
| 5719-66 | SL014977 | Q15884 | CI061 | family with sequence similarity 189, member A2 | 1.17 (1.09, 1.26) | 1.71e-05 | 0.93 (0.89, 0.96) | 2.35e-05 |
| 11416-23 | SL018669 | Q9UKA2 | FBXL4 | F-box and leucine-rich repeat protein 4 | 1.18 (1.10, 1.27) | 6.96e-06 | 0.93 (0.90, 0.96) | 2.20e-05 |
| 6991-24 | SL006795 | Q9Y231 | FUT9 | fucosyltransferase 9 (alpha (1,3) fucosyltransferase) | 1.16 (1.09, 1.24) | 1.37e-05 | 0.94 (0.91, 0.97) | 2.55e-04 |
| 7185-29 | SL006482 | P40197 | GPV | glycoprotein V (platelet) | 1.16 (1.09, 1.24) | 7.16e-06 | 0.95 (0.92, 0.98) | 4.86e-04 |
| 10760-107 | SL018367 | P48058 | GRIA4 | glutamate receptor, ionotrophic, AMPA 4 | 1.16 (1.09, 1.24) | 1.22e-05 | 0.93 (0.90, 0.96) | 2.26e-05 |
| 4237-70 | SL011770 | Q9UIC8 | LCMT1 | leucine carboxyl methyltransferase 1 | 1.19 (1.12, 1.28) | 4.78e-07 | 0.93 (0.90, 0.96) | 3.08e-05 |
| 8097-77 | SL018395 | Q5VXI9 | LIPN | lipase, family member N | 1.19 (1.11, 1.28) | 9.97e-07 | 0.93 (0.90, 0.96) | 3.00e-05 |
| 5004-69 | SL007453 | Q15759 | MK11 | mitogen-activated protein kinase 11 | 0.86 (0.80, 0.92) | 5.14e-06 | 1.07 (1.03, 1.1) | 6.34e-05 |
| 12891-1 | SL019924 | Q8IY33 | MILK2 | MICAL-like 2 | 1.17 (1.10, 1.26) | 7.70e-06 | 0.93 (0.90, 0.96) | 2.78e-05 |
| 9963-19 | SL019221 | Q9UN67 | PCDBA | protocadherin beta 10 | 1.18 (1.10, 1.27) | 1.55e-06 | 0.93 (0.90, 0.96) | 2.72e-05 |
| 4544-4 | SL004708 | P02775 | CTAP-III | pro-platelet basic protein (chemokine (C-X-C motif) ligand 7) | 1.17 (1.09, 1.25) | 5.87e-06 | 0.94 (0.91, 0.97) | 2.62e-04 |
| 3859-50 | SL010973 | P25786 | PSA1 | proteasome (prosome, macropain) subunit, alpha type, 1 | 1.19 (1.11, 1.29) | 3.58e-06 | 0.94 (0.90, 0.97) | 3.44e-04 |
| 9887-40 | SL019143 | Q7Z6E9 | RBBP6 | retinoblastoma binding protein 6 | 1.18 (1.10, 1.27) | 3.20e-06 | 0.94 (0.91, 0.97) | 1.08e-04 |
| 14670-1 | SL020187 | P12757 | SKIL | SKI-like oncogene | 1.18 (1.10, 1.27) | 6.44e-06 | 0.93 (0.90, 0.96) | 3.81e-05 |
| 8098-37 | SL011184 | P24557 | THAS | thromboxane A synthase 1 (platelet) | 1.19 (1.11, 1.28) | 5.05e-07 | 0.93 (0.9, 0.96) | 2.44e-05 |
| 9638-2 | SL019091 | None | TIGIT | T cell immunoreceptor with Ig and ITIM domains | 0.87 (0.81, 0.92) | 1.89e-05 | 1.06 (1.03, 1.09) | 3.59e-04 |
| 12742-160 | SL019870 | A6NDV4 | TMM8B | transmembrane protein 8B | 1.17 (1.10, 1.25) | 1.95e-06 | 0.93 (0.90, 0.96) | 2.87e-05 |
| 3403-1 | SL010617 | P20231 | TPSB2 | tryptase beta 2 (gene/pseudogene) | 0.87 (0.81, 0.92) | 1.29e-05 | 1.06 (1.03, 1.09) | 2.00e-04 |
| 8263-64 | SL018450 | Q8TAI1 | CR056 | chromosome 18 open reading frame 56 | 1.17 (1.09, 1.26) | 1.24e-05 | 0.93 (0.90, 0.96) | 5.35e-05 |
| 9215-117 | SL018947 | Q9UHP3 | UBP25 | ubiquitin specific peptidase 25 | 1.17 (1.09, 1.25) | 6.17e-06 | 0.93 (0.90, 0.96) | 2.67e-05 |
| 6290-3 | SL012625 | Q765I0 | UTS2B | urotensin 2 domain containing | 0.86 (0.81, 0.92) | 1.65e-05 | 1.05 (1.02, 1.09) | 1.67e-03 |
| 10023-32 | SL004071 | P11473 | VDR | vitamin D (1,25- dihydroxyvitamin D3) receptor | 1.19 (1.10, 1.28) | 1.50e-05 | 0.93 (0.90, 0.97) | 3.08e-04 |


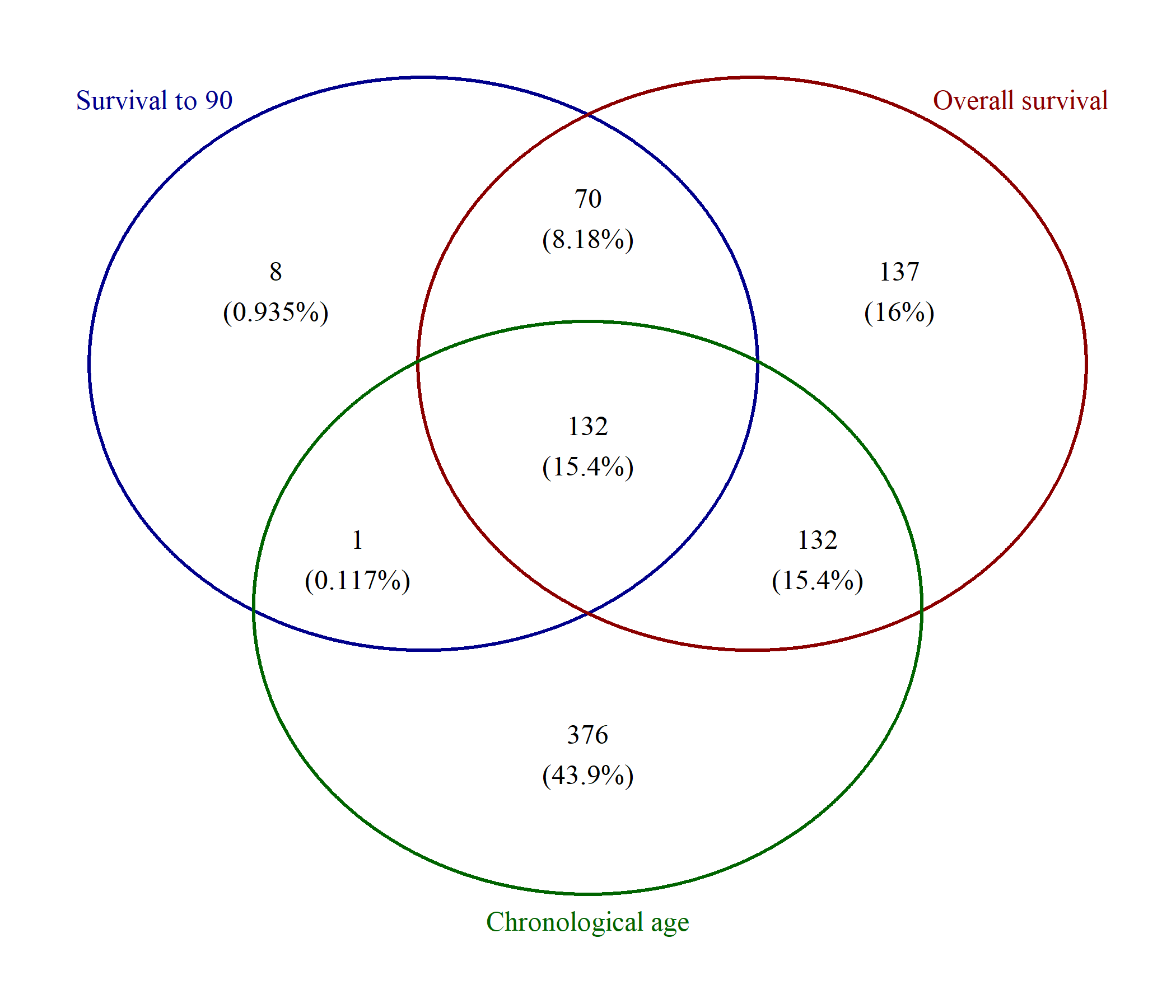


Figure S2. Venn diagram showing the difference of the significant proteins for chronological age, survival to 90, and overall survival in CHS.


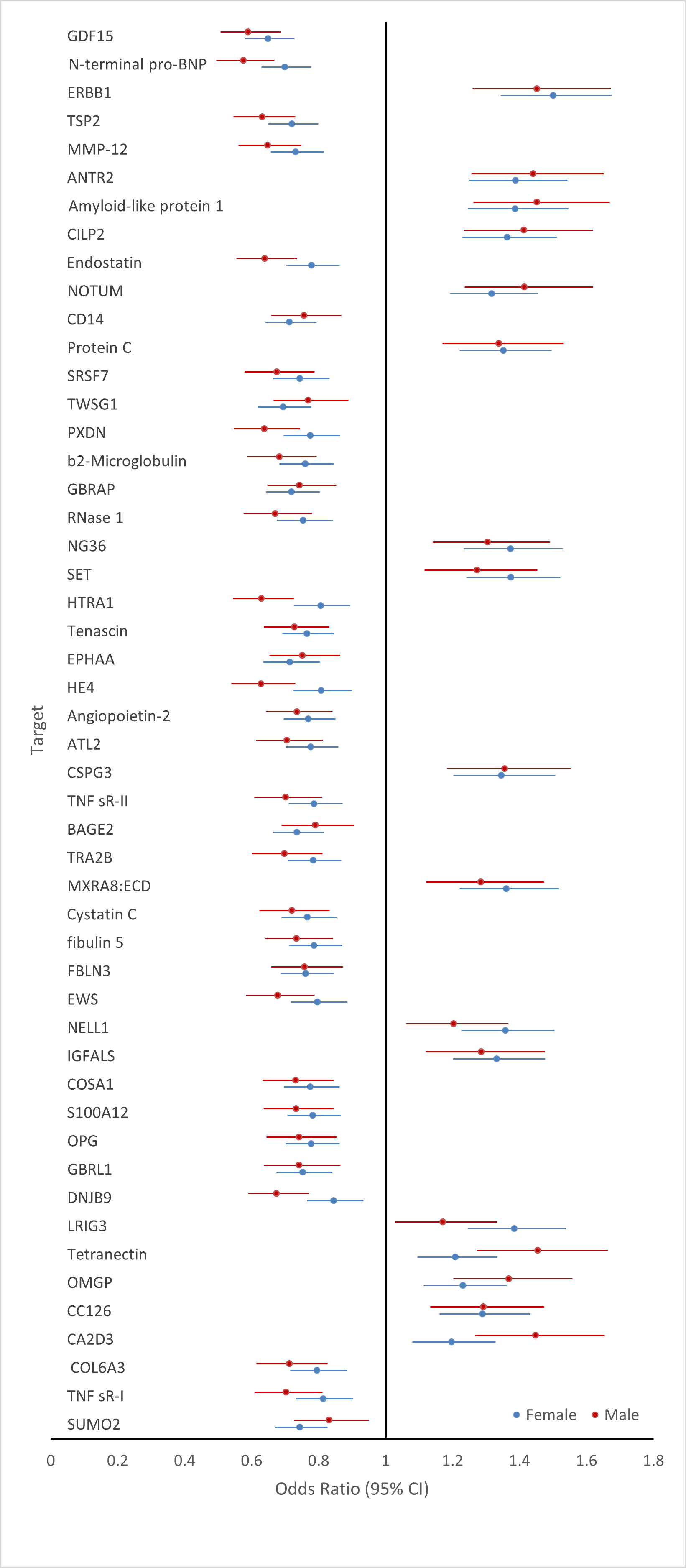


Figure S3. Sex-specific associations of top 50 proteins with survival to 90 in CHS

Table S5. Coefficients of the proteins chosen by LASSO (n=36).

| SeqId | SomaId | UniProt | Target | Target full name | Coefficients | Ranking* |
| --- | --- | --- | --- | --- | --- | --- |
| 7655-11 | SL002785 | P16860 | N-terminal pro-BNP | N-terminal pro-BNP | -0.23740 | 2 |
| 4374-45 | SL003869 | Q99988 | GDF-15 | Growth/differentiation factor 15 | -0.20536 | 1 |
| 4496-60 | SL000522 | P39900 | MMP-12 | Macrophage metalloelastase | -0.07922 | 5 |
| 15511-37 | SL008970 | O95502 | NPTXR | Neuronal pentraxin receptor | 0.07737 | 74 |
| 9216-100 | SL009948 | O15031 | PLXB2 | Plexin-B2 | -0.07641 | 105 |
| 7210-25 | SL004470 | P51693 | Amyloid-like protein 1 | Amyloid-like protein 1 | 0.07438 | 7 |
| 10521-10 | SL025939 | Q9BRK3 | MXRA8:ECD | Matrix-remodeling-associated protein 8:Extracellular domain | 0.07119 | 31 |
| 8969-49 | SL018823 | P08571 | CD14 | Monocyte differentiation antigen CD14 | -0.07084 | 11 |
| 2201-17 | SL000403 | P39060 | Endostatin | Endostatin | -0.06042 | 9 |
| 3322-52 | SL010464 | Q6UXM1 | LRIG3 | Leucine-rich repeats and immunoglobulin-like domains protein 3 | 0.05859 | 43 |
| 8841-65 | SL008847 | Q8IUL8 | CILP2 | Cartilage intermediate layer protein 2 | 0.05492 | 8 |
| 5852-6 | SL004783 | P80511 | S100A12 | Protein S100-A12 | -0.05298 | 39 |
| 9021-1 | SL010386 | Q96D42 | TIM-1 | Hepatitis A virus cellular receptor 1 | -0.05088 | 55 |
| 9348-1 | SL010648 | Q9NZP8 | C1RL1 | Complement C1r subcomponent-like protein | -0.04831 | 84 |
| 16607-78 | SL005572 | P06396 | Gelsolin | Gelsolin | 0.04701 | 62 |
| 8304-50 | SL002539 | O00300 | OPG | Tumor necrosis factor receptor superfamily member 11B | -0.04655 | 40 |
| 12630-8 | SL019787 | P53365 | ARFP2 | Arfaptin-2 | 0.04247 | 57 |
| 6036-78 | SL014294 | Q5JZY3 | EPHAA | Ephrin type-A receptor 10 | -0.04018 | 23 |
| 8252-2 | SL012580 | Q6P988 | NOTUM | Palmitoleoyl-protein carboxylesterase NOTUM | 0.03841 | 10 |
| 19251-56 | SL005269 | P10124 | Platelet proteoglycan | Serglycin | -0.03202 | 146 |
| 5843-60 | SL003542 | Q96KQ7 | NG36 | Histone-lysine N-methyltransferase EHMT2 | 0.02952 | 19 |
| 17729-20 | SL014981 | Q16763 | UBE2S | Ubiquitin-conjugating enzyme E2 S | -0.02890 | 137 |
| 3029-52 | SL005157 | Q9NNX6 | DC-SIGN | CD209 antigen | 0.02412 | 126 |
| 4297-62 | SL005115 | Q9HCB6 | Spondin-1 | Spondin-1 | -0.01736 | 101 |
| 19584-33 | SL003849 | P31371 | FGF9 | Fibroblast growth factor 9 | 0.01516 | 67 |
| 18339-207 | SL008674 | P49720 | PSB3 | Proteasome subunit beta type-3 | -0.01138 | 100 |
| 16908-5 | SL010377 | P23515 | OMGP | Oligodendrocyte-myelin glycoprotein | 0.00970 | 45 |
| 15573-110 | SL008782 | O14594 | CSPG3 | Neurocan core protein | 0.00939 | 27 |
| 19563-3 | SL008781 | Q9BYH1 | SEZ6L | Seizure 6-like protein | 0.00830 | 155 |
| 14227-21 | SL008040 | P14649 | MYL6B | Myosin light chain 6B | -0.00777 | 112 |
| 9525-1 | SL013388 | Q13308 | PTK7 | Inactive tyrosine-protein kinase 7 | -0.00630 | 151 |
| 6544-33 | SL012542 | Q92832 | NELL1 | Protein kinase C-binding protein NELL1 | 0.00512 | 36 |
| 5738-25 | SL003198 | P24821 | Tenascin | Tenascin | -0.00415 | 144 |
| 2677-1 | SL002644 | P00533 | ERBB1 | Epidermal growth factor receptor | 0.00190 | 3 |
| 8300-82 | SL025958 | O75381 | PEX14:N-term | Peroxisomal membrane protein PEX14:N-term | -0.00051 | 211 |
| 9580-5 | SL005798 | Q13753 | Laminin gamma-2 | Laminin subunit gamma-2 | -0.00040 | 164 |

Note: * The ranking (ordered by significance) of the associations with survival to 90. Coefficients were estimated using CHS data. Proteins marked in bold (n=27) were measured in AGES-Reykjavik and included in a validation model.

Table S6. Average causal mediation effect (ACME) and percentage mediated of 4 functional measurements for partially mediating the associations of proteins with survival to 90.

| SeqId | SomaId | UniProt | Target | Target full name | ACME (% mediated) | | | | Ranking* |
| --- | --- | --- | --- | --- | --- | --- | --- | --- | --- |
|  |  |  |  |  | Gait speed | Grip strength | DSST | MMSE |  |
| 4153-11 | SL018548 | P01011 | alpha-1-antichymotrypsin complex | Alpha-1-antichymotrypsin complex | -0.0065 (0.14) | -0.0020 (0.04) | -0.0058 (0.12) | -0.0026 (0.06) | 97 |
| 3079-62 | SL005152 | Q99969 | TIG2 | Retinoic acid receptor responder protein 2 | -0.0051 (0.10) | -0.0017 (0.03) | -0.0053 (0.10) | -0.0023 (0.05) | 71 |
| 3024-18 | SL000250 | P08697 | a2-Antiplasmin | Alpha-2-antiplasmin | 0.0043 (0.11) | 0.0017 (0.04) | 0.0058 (0.15) | 0.0031 (0.07) | 177 |
| 8841-65 | SL008847 | Q8IUL8 | CILP2 | Cartilage intermediate layer protein 2 | 0.0052 (0.08) | -0.0033 (-0.05) | 0.0097 (0.15) | 0.0035 (0.05) | 8 |
| 5456-59 | SL006694 | Q96KN2 | CNDP1 | Beta-Ala-His dipeptidase | 0.0048 (0.12) | 0.0011 (0.03) | 0.0069 (0.19) | 0.0037 (0.09) | 195 |
| 10702-1 | SL012521 | Q2UY09 | COSA1 | Collagen alpha-1(XXVIII) chain | -0.0053 (0.09) | -0.0024 (0.04) | -0.0036 (0.06) | -0.0012 (0.02) | 38 |
| 11214-40 | SL019363 | Q9UBS3 | DNJB9 | DnaJ homolog subfamily B member 9 | -0.0052 (0.09) | -0.0014 (0.02) | -0.0051 (0.09) | -0.0023 (0.04) | 42 |
| 2677-1 | SL002644 | P00533 | ERBB1 | Epidermal growth factor receptor | 0.0065 (0.08) | 0.0021 (0.02) | 0.0092 (0.11) | 0.0028 (0.03) | 3 |
| 15585-304 | SL004460 | Q9UBX5 | fibulin 5 | Fibulin-5 | -0.0046 (0.08) | -0.0009 (0.02) | -0.0048 (0.08) | -0.0019 (0.03) | 33 |
| 8265-225 | SL013037 | P42167 | LAP2B | Lamina-associated polypeptide 2, isoforms beta/gamma | 0.0044 (0.07) | 0.0020 (0.03) | 0.0043 (0.06) | 0.0021 (0.03) | 64 |
| 4496-60 | SL000522 | P39900 | MMP-12 | Macrophage metalloelastase | -0.0056 (0.07) | -0.0014 (0.02) | -0.0076 (0.10) | -0.0025 (0.03) | 5 |
| 6927-7 | SL017913 | P52848 | NDST1 | Bifunctional heparan sulfate N-deacetylase/N-sulfotransferase 1 | 0.0043 (0.08) | 0.0014 (0.03) | 0.0095 (0.17) | 0.0031 (0.05) | 127 |
| 2692-74 | SL002528 | P14555 | NPS-PLA2 | Phospholipase A2, membrane associated | -0.0068 (0.17) | -0.0023 (0.05) | -0.0066 (0.16) | -0.0024 (0.06) | 156 |
| 15304-1 | SL002648 | Q06141 | PAP1 | Regenerating islet-derived protein 3-alpha | -0.0050 (0.12) | -0.0020 (0.05) | -0.0058 (0.14) | -0.0026 (0.06) | 162 |
| 7957-2 | SL012750 | Q8WXD2 | SCG3 | Secretogranin-3 | -0.0041 (0.09) | -0.0021 (0.04) | -0.0043 (0.09) | -0.0024 (0.05) | 167 |
| 5364-7 | SL007336 | Q01105 | SET | Protein SET | 0.0051 (0.08) | 0.0015 (0.02) | 0.0085 (0.14) | 0.0028 (0.05) | 20 |
| 4297-62 | SL005115 | Q9HCB6 | Spondin-1 | Spondin-1 | -0.0038 (0.08) | -0.0018 (0.04) | -0.0076 (0.17) | -0.0029 (0.06) | 101 |
| 9021-1 | SL010386 | Q96D42 | TIM-1 | Hepatitis A virus cellular receptor 1 | -0.0045 (0.07) | -0.0021 (0.03) | -0.0069 (0.10) | -0.0019 (0.03) | 55 |
| 6291-55 | SL011368 | Q9BQT9 | Alcadein-beta | Calsyntenin-3 | -0.0035 (0.07) | -0.0005 (0.01) | -0.0052 (0.11) | 0.0013 (-0.03) | 107 |
| 7210-25 | SL004470 | P51693 | Amyloid-like protein 1 | Amyloid-like protein 1 | 0.0046 (0.11) | 0.0014 (0.03) | 0.0071 (0.17) | 0.0012 (0.03) | 7 |
| 10464-6 | SL008696 | Q9H6X2 | ANTR1 | Anthrax toxin receptor 1 | 0.0065 (0.13) | 0.0005 (0.01) | 0.0050 (0.10) | 0.0012 (0.02) | 78 |
| 15559-5 | SL011048 | P58335 | ANTR2 | Anthrax toxin receptor 2 | 0.0075 (0.10) | 0.0013 (0.02) | 0.0066 (0.09) | 0.0012 (0.02) | 6 |
| 6379-62 | SL012648 | Q86TH1 | ATL2 | ADAMTS-like protein 2 | -0.0049 (0.11) | -0.0010 (0.02) | -0.0057 (0.12) | -0.0011 (0.02) | 26 |
| 3485-28 | SL000283 | P61769 | b2-Microglobulin | Beta-2-microglobulin | -0.0053 (0.08) | -0.0018 (0.03) | -0.0037 (0.05) | -0.0008 (0.01) | 16 |
| 10832-24 | SL018597 | Q9UBX8 | B4GT6 | Beta-1,4-galactosyltransferase 6 | 0.0040 (0.10) | 0.0003 (0.01) | 0.0051 (0.12) | 0.0011 (0.03) | 200 |
| 16751-15 | SL002689 | P16860 | BNP | Natriuretic peptides B | -0.0032 (0.08) | -0.0019 (0.05) | -0.0038 (0.09) | -0.0016 (0.04) | 138 |
| 9348-1 | SL010648 | Q9NZP8 | C1RL1 | Complement C1r subcomponent-like protein | -0.0026 (0.06) | -0.0014 (0.03) | -0.0057 (0.15) | -0.0005 (0.01) | 84 |
| 13950-9 | SL015375 | Q9Y5P4 | C43BP | Collagen type IV alpha-3-binding protein | -0.0039 (0.09) | -0.0008 (0.02) | -0.0065 (0.15) | 0.0012 (-0.03) | 103 |
| 8885-6 | SL018710 | Q8IZS8 | CA2D3 | Voltage-dependent calcium channel subunit alpha-2/delta-3 | 0.0058 (0.08) | 0.0009 (0.01) | 0.0061 (0.09) | 0.0019 (0.03) | 47 |
| 5688-65 | SL012852 | Q9NTU7 | CBLN4 | Cerebellin-4 | 0.0031 (0.08) | 0.0017 (0.04) | 0.0024 (0.06) | 0.0015 (0.03) | 145 |
| 5717-2 | SL004657 | O75339 | CILP | Cartilage intermediate layer protein 1 | -0.0035 (0.07) | -0.0005 (0.01) | -0.0050 (0.10) | 0.0020 (-0.04) | 60 |
| 17327-3 | SL017270 | Q9BT09 | CNPY3 | Protein canopy homolog 3 | -0.0042 (0.11) | -0.0012 (0.03) | -0.0050 (0.13) | -0.0005 (0.01) | 161 |
| 6570-1 | SL017501 | Q5TAT6 | CODA1 | Collagen alpha-1(XIII) chain | -0.0038 (0.09) | -0.0006 (0.01) | -0.0045 (0.11) | -0.0011 (0.02) | 114 |
| 11196-31 | SL004928 | P12111 | Collagen alpha-3(VI) | Collagen alpha-3(VI) chain | -0.0052 (0.09) | -0.0023 (0.04) | -0.0039 (0.06) | -0.0014 (0.02) | 48 |
| 15573-110 | SL008782 | O14594 | CSPG3 | Neurocan core protein | 0.0042 (0.07) | 0.0014 (0.02) | 0.0096 (0.15) | 0.0017 (0.03) | 27 |
| 3029-52 | SL005157 | Q9NNX6 | DC-SIGN | CD209 antigen | 0.0036 (0.09) | 0.0007 (0.02) | 0.0050 (0.12) | 0.0021 (0.05) | 126 |
| 4834-61 | SL002654 | P29317 | Epithelial cell kinase | Ephrin type-A receptor 2 | -0.0049 (0.11) | -0.0019 (0.04) | -0.0023 (0.05) | -0.0015 (0.03) | 93 |
| 6284-7 | SL012605 | Q6UX46 | F150B | ALK and LTK ligand 2 | -0.0031 (0.08) | -0.0007 (0.02) | -0.0061 (0.17) | -0.0006 (0.01) | 96 |
| 8480-29 | SL006527 | Q12805 | FBLN3 | EGF-containing fibulin-like extracellular matrix protein 1 | -0.0056 (0.12) | -0.0023 (0.05) | -0.0039 (0.08) | -0.0020 (0.04) | 34 |
| 19584-33 | SL003849 | P31371 | FGF9 | Fibroblast growth factor 9 | 0.0034 (0.07) | -0.0000 (-0.00) | 0.0054 (0.11) | 0.0015 (0.03) | 67 |
| 3438-10 | SL009324 | O95633 | FSTL3 | Follistatin-related protein 3 | -0.0048 (0.09) | -0.0018 (0.03) | -0.0031 (0.06) | -0.0006 (0.01) | 76 |
| 15522-2 | SL008305 | Q9H4G4 | GAPR1 | Golgi-associated plant pathogenesis-related protein 1 | 0.0055 (0.11) | 0.0009 (0.02) | 0.0067 (0.14) | 0.0019 (0.04) | 66 |
| 17735-130 | SL020978 | O95166 | GBRAP | Gamma-aminobutyric acid receptor-associated protein | -0.0054 (0.08) | -0.0015 (0.02) | -0.0065 (0.10) | -0.0001 (0.00) | 17 |
| 12661-44 | SL019788 | Q9H0R8 | GBRL1 | Gamma-aminobutyric acid receptor-associated protein-like 1 | -0.0048 (0.08) | -0.0018 (0.03) | -0.0061 (0.11) | -0.0002 (0.00) | 41 |
| 19238-12 | SL008477 | P15104 | GLNA | Glutamine synthetase | -0.0043 (0.10) | -0.0004 (0.01) | -0.0053 (0.14) | 0.0006 (-0.01) | 119 |
| 18896-23 | SL021212 | Q8IZP7 | H6ST3 | Heparan-sulfate 6-O-sulfotransferase 3 | 0.0031 (0.06) | 0.0006 (0.01) | 0.0066 (0.14) | 0.0012 (0.02) | 99 |
| 16758-96 | SL013046 | P51858 | HDGF | Hepatoma-derived growth factor | -0.0042 (0.09) | -0.0010 (0.02) | -0.0055 (0.12) | -0.0005 (0.01) | 70 |
| 8953-47 | SL013046 | P51858 | HDGF | Hepatoma-derived growth factor | 0.0056 (0.10) | 0.0015 (0.03) | 0.0050 (0.09) | 0.0012 (0.02) | 159 |
| 15594-47 | SL008268 | Q92743 | HTRA1 | Serine protease HTRA1 | -0.0042 (0.07) | -0.0024 (0.04) | -0.0064 (0.10) | -0.0009 (0.01) | 21 |
| 8469-41 | SL000466 | P18065 | IGFBP-2 | Insulin-like growth factor-binding protein 2 | -0.0063 (0.12) | -0.0023 (0.04) | -0.0044 (0.08) | -0.0013 (0.02) | 173 |
| 5353-89 | SL001990 | P18510 | IL-1Ra | Interleukin-1 receptor antagonist protein | -0.0036 (0.09) | -0.0011 (0.03) | -0.0043 (0.11) | -0.0001 (0.00) | 179 |
| 8484-24 | SL000498 | P41159 | Leptin | Leptin | -0.0053 (0.09) | -0.0023 (0.04) | -0.0060 (0.10) | -0.0014 (0.02) | 180 |
| 3322-52 | SL010464 | Q6UXM1 | LRIG3 | Leucine-rich repeats and immunoglobulin-like domains protein 3 | 0.0036 (0.07) | 0.0004 (0.01) | 0.0073 (0.14) | 0.0011 (0.02) | 43 |
| 15533-97 | SL004580 | P21757 | Macrophage scavenger receptor | Macrophage scavenger receptor types I and II | -0.0039 (0.10) | -0.0005 (0.01) | -0.0068 (0.16) | -0.0012 (0.03) | 153 |
| 4374-45 | SL003869 | Q99988 | MIC-1 | Growth/differentiation factor 15 | -0.0062 (0.06) | -0.0022 (0.02) | -0.0063 (0.06) | -0.0014 (0.01) | 1 |
| 2789-26 | SL000525 | P09237 | MMP-7 | Matrilysin | -0.0047 (0.13) | 0.0003 (-0.01) | -0.0022 (0.06) | -0.0021 (0.05) | 202 |
| 10521-10 | SL017989 | Q9BRK3 | MXRA8 | Matrix-remodeling-associated protein 8 | 0.0061 (0.10) | -0.0003 (-0.00) | 0.0065 (0.11) | 0.0015 (0.02) | 31 |
| 14227-21 | SL008040 | P14649 | MYL6B | Myosin light chain 6B | -0.0047 (0.10) | -0.0007 (0.01) | -0.0063 (0.14) | -0.0014 (0.03) | 112 |
| 6544-33 | SL012542 | Q92832 | NELL1 | Protein kinase C-binding protein NELL1 | 0.0039 (0.07) | 0.0008 (0.01) | 0.0050 (0.09) | 0.0007 (0.01) | 36 |
| 5843-60 | SL003542 | Q96KQ7 | NG36 | Histone-lysine N-methyltransferase EHMT2 | 0.0051 (0.08) | 0.0007 (0.01) | 0.0051 (0.08) | 0.0016 (0.02) | 19 |
| 15511-37 | SL008970 | O95502 | NPTXR | Neuronal pentraxin receptor | 0.0034 (0.06) | -0.0001 (-0.00) | 0.0054 (0.11) | 0.0020 (0.04) | 74 |
| 5542-22 | SL006397 | O14786 | NRP1 | Neuropilin-1 | -0.0030 (0.07) | -0.0012 (0.03) | -0.0055 (0.14) | -0.0009 (0.02) | 204 |
| 7655-11 | SL002785 | P16860 | N-terminal pro-BNP | N-terminal pro-BNP | 0.0044 (0.10) | 0.0014 (0.03) | 0.0055 (0.13) | 0.0012 (0.03) | 2 |
| 13565-2 | SL003760 | Q08999 | p130 | Retinoblastoma-like protein 2 | -0.0044 (0.11) | -0.0012 (0.03) | -0.0043 (0.10) | -0.0019 (0.05) | 172 |
| 4152-58 | SL000545 | P03952 | Prekallikrein | Plasma kallikrein | 0.0049 (0.12) | 0.0014 (0.03) | 0.0054 (0.14) | 0.0024 (0.06) | 182 |
| 2961-1 | SL000048 | P04070 | Protein C | Vitamin K-dependent protein C | 0.0041 (0.06) | 0.0014 (0.02) | 0.0076 (0.12) | 0.0009 (0.01) | 12 |
| 17176-13 | SL014348 | O75475 | PSIP1 | PC4 and SFRS1-interacting protein | -0.0036 (0.07) | -0.0005 (0.01) | -0.0046 (0.10) | 0.0002 (-0.00) | 82 |
| 14037-18 | SL013273 | Q9H6Z4 | RANB3 | Ran-binding protein 3 | -0.0048 (0.09) | -0.0016 (0.03) | -0.0043 (0.08) | 0.0006 (-0.01) | 53 |
| 7211-2 | SL005355 | P07998 | Rnase 1 | Ribonuclease pancreatic | 0.0042 (0.06) | 0.0011 (0.01) | 0.0073 (0.10) | 0.0024 (0.03) | 18 |
| 7861-9 | SL008955 | Q01974 | ROR2 | Tyrosine-protein kinase transmembrane receptor ROR2 | -0.0038 (0.04) | -0.0013 (0.01) | -0.0054 (0.06) | -0.0019 (0.02) | 143 |
| 10490-3 | SL011102 | P04843 | RPN1 | Dolichyl-diphosphooligosaccharide--protein glycosyltransferase subunit 1 | -0.0030 (0.07) | -0.0014 (0.03) | -0.0050 (0.12) | 0.0006 (-0.01) | 115 |
| 5852-6 | SL004783 | P80511 | S100A12 | Protein S100-A12 | -0.0038 (0.07) | -0.0019 (0.03) | -0.0037 (0.07) | -0.0012 (0.02) | 39 |
| 15441-6 | SL008548 | P17900 | SAP3 | Ganglioside GM2 activator | -0.0057 (0.13) | -0.0025 (0.05) | -0.0028 (0.06) | -0.0020 (0.04) | 116 |
| 5029-3 | SL006528 | Q12884 | SEPR | Prolyl endopeptidase FAP | 0.0049 (0.10) | 0.0007 (0.01) | 0.0038 (0.07) | 0.0004 (0.01) | 61 |
| 5129-12 | SL005221 | Q14162 | SREC-I | Scavenger receptor class F member 1 | -0.0047 (0.12) | -0.0006 (0.01) | -0.0057 (0.14) | 0.0007 (-0.02) | 154 |
| 9266-1 | SL004968 | Q9NP99 | sTREM-1 | Triggering receptor expressed on myeloid cells 1 | -0.0053 (0.08) | -0.0018 (0.03) | -0.0047 (0.07) | -0.0010 (0.01) | 122 |
| 10418-36 | SL011111 | Q86Y82 | STX12 | Syntaxin-12 | -0.0034 (0.09) | -0.0007 (0.02) | -0.0045 (0.12) | 0.0009 (-0.02) | 210 |
| 10903-50 | SL017987 | Q9UNK0 | STX8 | Syntaxin-8 | -0.0039 (0.08) | -0.0009 (0.02) | -0.0055 (0.11) | 0.0012 (-0.02) | 59 |
| 19555-1 | SL013060 | P61956 | SUMO2 | Small ubiquitin-related modifier 2 | -0.0036 (0.07) | -0.0012 (0.02) | -0.0060 (0.11) | 0.0012 (-0.02) | 50 |
| 11109-56 | SL012540 | Q4LDE5 | SVEP1 | Sushi, von Willebrand factor type A, EGF and pentraxin domain-containing protein 1 | -0.0062 (0.14) | -0.0025 (0.06) | -0.0053 (0.13) | -0.0023 (0.05) | 118 |
| 11178-21 | SL012540 | Q4LDE5 | SVEP1 | Sushi, von Willebrand factor type A, EGF and pentraxin domain-containing protein 1 | -0.0058 (0.11) | -0.0024 (0.05) | -0.0049 (0.10) | -0.0025 (0.05) | 54 |
| 4157-2 | SL000586 | P00734 | Thrombin | Thrombin | 0.0040 (0.07) | 0.0007 (0.01) | 0.0042 (0.08) | 0.0007 (0.01) | 51 |
| 3152-57 | SL001800 | P20333 | TNF sR-II | Tumor necrosis factor receptor superfamily member 1B | -0.0058 (0.10) | -0.0022 (0.04) | -0.0040 (0.07) | -0.0011 (0.02) | 28 |
| 8368-102 | SL001800 | P20333 | TNF sR-II | Tumor necrosis factor receptor superfamily member 1B | -0.0051 (0.09) | -0.0016 (0.03) | -0.0059 (0.10) | -0.0021 (0.04) | 52 |
| 11361-73 | SL000155 | P19971 | TP | Thymidine phosphorylase | -0.0041 (0.10) | 0.0002 (-0.00) | -0.0061 (0.15) | 0.0007 (-0.02) | 130 |
| 3339-33 | SL007206 | P35442 | TSP2 | Thrombospondin-2 | -0.0044 (0.06) | -0.0018 (0.02) | -0.0052 (0.07) | -0.0018 (0.02) | 4 |
| 11212-7 | SL008805 | Q8NBS9 | TXND5 | Thioredoxin domain-containing protein 5 | -0.0038 (0.08) | -0.0016 (0.03) | -0.0069 (0.15) | -0.0010 (0.02) | 90 |
| 3651-50 | SL003201 | P35968 | VEGF sR2 | Vascular endothelial growth factor receptor 2 | 0.0039 (0.09) | 0.0009 (0.02) | 0.0063 (0.15) | 0.0012 (0.03) | 129 |

Notes: This shows those protein associations mediated by at least two functional measurements (n=88). Green: negative ACME; Red: positive ACME; Blank: non-significant ACME. * The ranking (ordered by significance) of the associations with survival to 90
